# Insights into pancreatic β cell energy metabolism using rodent β cell models

**DOI:** 10.12688/wellcomeopenres.10535.3

**Published:** 2019-09-25

**Authors:** Karl J Morten, Michelle Potter, Luned Badder, Pamela Sivathondan, Rebecca Dragovic, Abigale Neumann, James Gavin, Roshan Shrestha, Svetlana Reilly, Kanchan Phadwal, Tiffany A. Lodge, Angela Borzychowski, Sharon Cookson, Corey Mitchell, Alireza Morovat, Anna Katharina Simon, Johanna Uusimaa, James Hynes, Joanna Poulton

**Affiliations:** 1 Nuffield Department of Obstetrics & Gynaecology, The Women’s Centre, University of Oxford, John Radcliffe Hospital, Oxford, UK; 2 Department of Cardiovascular Medicine, John Radcliffe Hospital, Oxford, UK; 3 BRC Translational Immunology Lab, NIHR, Nuffield Department of Medicine, University of Oxford, John Radcliffe Hospital, Oxford, UK; 4 Institute of Cellular Medicine, Haematological Sciences, Medical School, Newcastle University, Newcastle upon Tyne, UK; 5 Clinical Biochemistry, John Radcliffe Hospital, Oxford, UK; 6 Kennedy Institute of Rheumatology, University of Oxford, Oxford, UK; 7 Department of Paediatrics, University of Oulu, Oulu, Finland; 8 Luxcel BioSciences Ltd, BioInnovation Centre, University College Cork, Cork, Ireland

**Keywords:** beta-cell, oxidative phosphorylation, reactive oxygen species, superoxide, mitochondria, insulin secretion

## Abstract

***Background**: *Mitochondrial diabetes is primarily caused by β-cell failure, a cell type whose unique properties are important in pathogenesis.

***Methods**: *By reducing glucose, we induced energetic stress in two rodent β-cell models to assess effects on cellular function.

***Results**: *Culturing rat insulin-secreting INS-1 cells in low glucose conditions caused a rapid reduction in whole cell respiration, associated with elevated mitochondrial reactive oxygen species production, and an altered glucose-stimulated insulin secretion profile. Prolonged exposure to reduced glucose directly impaired mitochondrial function and reduced autophagy.

***Conclusions**: *Insulinoma cell lines have a very different bioenergetic profile to many other cell lines and provide a useful model of mechanisms affecting β-cell mitochondrial function.

## Abbreviations

Reactive oxygen species (ROS), uncoupling protein 2 (UCP2), oxidative phosphorylation (OXPHOS), photomultiplier tubes (PMT), isolation buffer (ISO), respiratory control ratio (RCR), 2’-7’-dichlorofluoroscein diacetate (H _2_DCFDA,SE), Glucose stimulated insulin secretion (GSIS), endoplasmic reticulum (ER).

## Introduction

Mitochondria play a key role in glucose homeostasis ^1, 2^. Maternally inherited diabetes is caused by a mtDNA mutation with a population prevalence of 1 in 300 ^3^, affecting up to 1% of patients with diabetes ^4, 5^ and often going unrecognized by clinicians. Mitochondrial dysfunction in highly metabolically active pancreatic islets results in abnormal β-cell function linked to insulin deficiency (reviewed in 6). In patients whose diabetes is associated with mitochondrial DNA mutations, reduced β-cell mass may result from islet hypoplasia ^7^ or apoptosis ^8^, although the latter has never been demonstrated *in vivo* or *post mortem* tissue ^9^. The limited *post mortem* studies suggest that the mutant load of the pathogenic 3243 A-G mtDNA mutation in t-RNA leucine is low in the pancreas compared to other affected tissues, 30% contrasting with >70% in other tissues ^10^. This observation suggests that beta cells with high mutant load are likely to fail, and this is linked to reduced β-cell mass. In other affected tissues in patients carrying mtDNA mutations, mutant load is clearly linked to mitochondrial dysfunction ^11^.

The lack of a human beta cell model has made studying mitochondrial associated β-cell dysfunction *in vitro* challenging. Much of the relevant data comes from models based on other cell types, including cancer cell derived cybrids ^12^ and more recently neurons derived from induced pluripotent stem cells (IPS) cells ^13^. The specialized nature of the pancreatic β-cell, as a glucose sensor, supported by multiple specialized pathways of intermediary metabolism, makes comparison with other cell models particularly problematic. In the β-cell, mitochondria appear pivotal in regulating glucose stimulated insulin secretion (GSIS). Increasing glucose uptake not only increases ATP generation, but also activates mitochondrial metabolic pathways ^14^. Although metabolism and mitochondrial function play a role in driving insulin secretion under raised glucose conditions, it remains unclear which of the cellular signaling pathways involved is the key driver of the process. Reactive oxygen species (ROS) are not always deleterious, but are essential components of biological processes ^15, 16^. For instance, plasma membrane K _ATP_ channels can be inhibited either by elevated levels of ATP ^17^ or by raised levels of mitochondrial ROS without raised ATP levels ^18^. Both processes are able to drive insulin secretion via changes in cellular calcium influx.

The pancreatic β-cell mitochondria are compact and constantly involved in fusion and fission activity, which is altered depending upon nutrient exposure ^19^. Mitochondrial membrane potential within the mitochondrial network also varies with substrate exposure, becoming more heterogeneous following a low glucose or high glucose plus lipid challenge ^20^. Autophagy is essential for β-cell quality control; specific disruption of the autophagy process results in swollen mitochondria, high levels of ubiquitination and distention of the endoplasmic reticulum (ER) ^21, 22^. In rat insulin-secreting INS-1 cells, autophagy has been shown to be involved in the removal of dysfunctional mitochondria with low mitochondrial membrane potential following mitochondrial fission ^23^. Work by Affourtitt *et al.* ^24^, showed that the β-cell appears to have “metabolic safety valves” in place. For example, the mitochondrial uncoupling protein UCP2 seems to prevent over-stimulation of oxidative phosphorylation (OXPHOS) and excessive ROS production ^25^ during periods of high metabolic activity and insulin secretion. Hence, predicting how mitochondrial dysfunction associated with a mutation or substrate supply will impact on a β-cell is difficult. Common variants of mtDNA associated with type 2 diabetes have been identified and increase disease susceptibility in a wide range of populations ^26^. Studying the functional effects of these variants in a human β-cell model is not currently possible. We therefore tested rodent insulinoma models to determine whether they are suitable for studying the effects of mitochondrial dysfunction *in vitro*. An ideal *in vitro* β-cell model for studying mitochondrial dysfunction should demonstrate that GSIS is strongly linked to increased mitochondrial respiration as glucose levels are raised, linking increase energy metabolism to insulin secretion. Models that use β-cells derived from IPS cells are not yet robust. In this study we use two rodent β-cell lines INS-1 and MIN-6 to investigate the effect of glucose exposure on cell viability, mitochondrial function, GSIS and autophagy.

## Methods

### Chemicals

Unless otherwise stated, all chemicals used in this study were obtained from Sigma, UK.

### Cell lines

INS-1 cells were cultured in RPMI 1640 media (11mM glucose; Invitrogen), containing 15% fetal calf serum, 25mM Hepes, 50µM β-mercaptoethanol, 2mM L-glutamine, 100µg/ml streptomycin and 100U/ml penicillin. MIN-6 cells were cultured in DMEM media (25mM glucose; Invitrogen), containing 15% fetal calf serum, 1mM pyruvate, L-Glutamax, 50µM β-mercaptoethanol, 100µg/ml streptomycin and 100U/ml penicillin. Both cell lines were maintained under normoxic conditions in air plus 5% CO _2_ at 37°C. The data presented for both cell lines was accumulated from cultures between passages P22-P40. Glucose free media used in subsequent experiments for the two lines was identical to the standard culture media, except for the glucose free DMEM, which did not contain pyruvate. Glucose was then added at different concentrations depending upon the experiment. The MIN-6 and INS-1 cells were provided by Professor Patrik Rorsman (OCDEM, University of Oxford). MIN-6 and INS-1 cells were initially derived by Professors Miyazaki and Wolheim.

We also used HepG2 and U87MG cells (obtained from ATCC) for comparison as they show a reduction in mitochondrial respiration as glucose levels are raised ^27^. HepG2 and U87MG lines were cultured in DMEM media (25mM glucose), containing 10% fetal calf serum, 1mM pyruvate, 2mM L-glutamine, 100µg/ml streptomycin and 100 U/ml penicillin. For whole cell respiration and flow cytometry assays, which require cells to be in suspension, we used Accutase (PAA), a formulated mixture of digestive enzymes, to release cells from the tissue culture flasks. Several studies have shown Accutase to be less damaging to cells than trypsin ^28, 29^.

### Glucose stimulated insulin secretion (GSIS) assay

Initial experiments to demonstrate that INS-1 and MIN-6 cells show a GSIS response were carried out by culturing cells in 6 well plates. To assess the impact of a one hour reduced glucose challenge on the GSIS response of INS-1 cells, experiments were carried out as follows; INS-1 cells (100,000 per well) were cultured for 4 days in 12 well plates (Nunc). Prior to carrying out the GSIS assay, one of the plates was pre-incubated in RPMI 1640 media containing 1mM glucose for one hour. Media was then removed from both plates and cells were washed in pre-warmed Krebs Ringer Buffer (modified KRB: 2.54mM CaCl _2_, 1.199mM KH _2_PO _4_, 4.64mM KCL, 25mM NaHCO _3_, 1.19mM MgSO _4_, 10mM HEPES [pH 7.4]). 2ml of pre-warmed KRB containing 1, 5, 10 or 15mM glucose was added and incubated for 30 minutes. Each well was aspirated and replaced with 2ml of KRB containing the same concentrations of glucose and incubated for another 30 minutes. The supernatant was collected and assayed for insulin using a mouse insulin ELISA (Mercodia; catalog no., 10-1247-01-10). GSIS insulin secretion experiments were also carried out without the one hour pre-incubation in RPMI 1640 containing 1mM glucose.

### Assessment of cell growth

Cell growth/number under different growth conditions was assessed by determining cell number using a haemocytometer.

### Media glucose and lactate levels

Glucose was measured using the glucose oxidase, and lactate by the lactate oxidase assay on a Siemens ADVIA 2400 chemistry analyser. These are standard test carried out in NHS labs based on the hexokinase method (Glucose) and a method for lactate ^30, 31^.

### Mitochondrial and cellular ROS measurements

*MitoSox and flow cytometry:* MitoSox red (Invitrogen), a derivative of dihydroethidium, which is taken up by mitochondria in whole cells, was used to measure cellular mitochondrial ROS levels ^32, 33^. INS-1 and MIN-6 cells were grown in 175 cm ^2^ flasks under standard tissue culture conditions. Cells were harvested using Accutase and washed with PBS.

*Determination of non-saturating doses of MitoSox:* The MitoSox measurement of mitochondrial ROS relies upon the formation of the MitoSox/ROS product binding to mtDNA to produce mitochondrial fluorescence. Previous studies have shown that MitoSox labeling doses must be carefully titrated to avoid saturation of the dye (i.e. fluorescence rapidly reaches a maximum at high dye concentrations due to saturation of all of the available mtDNA) ^32^. As mtDNA levels are likely to be limiting in this reaction, we initially determined the concentration of MitoSox, which showed a linear increase in mitochondrial superoxide over a 3–4 hour period. In total, 100,000 cells for each cell line were incubated with 10nM, 100nM, 500nM, 1µM and 5µM MitoSox in DMEM (25mM glucose)/RPMI (11mM glucose) for 10 minutes. Cells were pelleted and washed twice in PBS before incubation in DMEM/RPMI containing 0mM glucose. Previous experiments had shown that 0mM glucose generated the highest amount of mitochondrial ROS compared to higher glucose incubations. As shown in Supplementary Figure 1, both cell lines show a non-saturating mitochondrial ROS production when cells are incubated for 10 minutes in media containing 5µM MitoSox prior to exposure to low glucose. Exposure to lower doses of MitoSox for 10 minutes did not detect significant levels of mitochondrial ROS in this system.

For glucose experiments, MIN-6 and INS-1 cells were grown under standard tissue culture conditions until 80% confluent. Cells were harvested and incubated with 5µM MitoSox for 10 minutes in standard tissue culture conditions. Following MitoSox staining, cells were washed with PBS and then split into six aliquots and incubated with different concentrations of glucose (0mm, 1mM, 2.5mM, 5mM, 10mM and 20mM). Cells were taken from the various treatments for analysis by flow cytometry at different time points. All samples were analyzed using a BD LSRII flow cytometer (BD Biosciences, Oxford, UK). MitoSox Red was excited using a 488nm laser and emission was detected using a bandpass filter of 575/26. Single cells were gated and 10,000 total cell events were collected. Data was analyzed using BD FACS Diva 5.0 software and mean fluorescence intensity (MFI) was compared. Under these conditions both the mitochondrial superoxide specific 2-hydroxyethidium (2-OH-E ^+^) and ethidium cation (E ^+^) are detected.

*Measuring mitochondrial ROS and cellular peroxide generation during low glucose exposure:* Cells were cultured as above, harvested using Accutase, and re-suspended in 8ml of DMEM (25mM glucose, MIN-6) or RPMI (11mM glucose, INS-1), containing a non-saturating concentration of MitoSox (5µM) and incubated for 10 minutes at 37°C. Additionally, cells were also incubated in standard cell culture media, containing the 2’7-dichlorofluoroscein derivative H2DCFDA,SE (Oxyburst Green) at a concentration of 10µm for 30 minutes at 37°C . Cells were pelleted and washed twice in PBS. Approximately 100,000 cells were then aliquoted into 1.5 ml Eppendorf tubes, pelleted and re-suspended in media containing the various treatment regimens and incubated at 37°C under standard tissue culture conditions (5% CO _2_, 20% O _2_). Holes were made in the lids of each tube to allow media equilibration with oxygen and 5% CO _2_. Following incubation for 60 minutes, cells were pelleted and re-suspended in PBS containing 0.5% FCS and kept on ice prior to flow cytometric analysis. The Oxyburst Green signal was analyzed using Beckman Coulter Epics Altra flow cytometer. In total, 10,000 events were collected and data was analyzed by Beckman Coulter Expo 32 software Photomultiplier tubes (PMTs), which collect fluorescent light at 525nm. Cell populations were initially identified on the basis of size (forward scatter) versus granularity (side scatter) and gated on these physical characteristics.

*Determination of whole cell superoxide levels using dihydroethidium (DHE) and high performance liquid chromatography (HPLC) analysis:* Measurement of 2-hydroxyethidium (2-OHE ^+^) formation by HPLC was used to detect cellular superoxide production, using methods adapted from those described previously ^34^. Cells were harvested with Accutase, washed once in PBS and incubated in standard tissue culture media containing DH (25µmol/l) for 20 minutes at 37°C. The cells were pelleted by centrifugation at 1,100xg for 3 minutes, washed twice in PBS and incubated in the dark at 37°C in media containing 1mM, 2.5mM, 5mM, 10mM or 20mM glucose for 3 hours. Cells were then pelleted, washed once in PBS and snap frozen in liquid nitrogen prior to storage at -80°C until required. The cells were lysed in ice-cold methanol. Hydrochloric acid (100mM) was added (1:1 v/v) prior to loading into the HPLC autosampler for analysis. All the samples were protected from light at all times. Separation of DHE, 2-OHE, and ethidium was performed using a gradient HPLC system (Jasco LTD, UK, Model 542) with an ODS3 reverse phase column (250 mm, 4.5 mm; Hichrom) and quantified using a fluorescence detector set at 510 nm (excitation) and 595 nm (emission). All results were subtracted with the negative (non-treated with DHE) control.

### Whole cell oxygen consumption measurements

*Oxygen electrode:* Whole cell oxygen consumption was determined using a Clark type oxygen electrode (Hansatech). Cells were incubated under the appropriate conditions, detached with Accutase, washed in PBS and re-suspended in glucose free RPMI/DMEM. Respiration rates were determined when cells were respiring at their maximal rates and expressed in nmoles oxygen consumed per minute.

*MitoXpress probes to detect oxygen consumption rates:* In order to assess the immediate effect of reduced levels of glucose on oxygen consumption rates, the MitoXpress Xtra system from Luxcel Biosciences was used. The assays were carried out according to the manufacturer’s protocol using Black 96 well plates with clear bottoms (BD Falcon) and the assays read using a FLUOstar Omega plate reader (BMG LABTECH Ltd). Integration start times of 30µs and 70µs were used with a measurement integration time of 30µs. The data was processed using the script mode function on the BMG FLUOstar Omega reader. Oxygen consumption rates were measured in suspension in media containing 0mM, 1mM, 2.5mM, 5mM, 10mM or 15mM glucose: for INS-1 and HepG2 cell lines, cells were grown in standard cell culture media, then washed with PBS and 100,000 cells were incubated per well in 150µl of media containing different concentrations of glucose. Using the MitoXpress Xtra assay, the maximal rate of oxygen consumption was determined by calculating the relative rate of oxygen consumed during the linear phase of the assay. Results are expressed as the change in relative fluorescence units (RFU)/min/100,000 cells. Supplementary Figure 2 gives an example of the oxygen consumption rate calculation. Previous studies have directly compared the rates of mitochondrial respiration, obtained with the MitoXpress probe and a Clark electrode approach. When assays are carried out under the same oxygen concentrations (20% oxygen) comparable rates for the removal of oxygen were obtained ^35^. The mitochondrial uncoupler FCCP was used to give an indication of the maximal possible rates of respiration.

Although, the Seahorse XF Analyzer (Agilent) system has a higher publication profile, the MitoXpress Luxcel probe systems to measure O _2_ consumption and extracellular acidification are becoming increasing adopted by many researchers ^36– 39^. This is because it has significant advantages over the Seahorse system including compatibility with many plate readers, multiplexing and when using the MitoXpress intra probe, experiments can be run over several days.

Here we also carried out assays on attached cells using the Luxcel MitoXpress Intra probe, to further compare the oxygen utilization profile of INS-1 cells to other tumour cell lines under different oxygen concentrations. Using this probe (see Supplementary figures: Figure 3 for a typical intracellular O2 experiment) intracellular O _2_ levels can be assessed in cells in real time over prolonged periods (i.e. hours or days). Using a plate reader such as the BMG Omega or Clariostar drugs and environmental conditions such as O _2_ can be varied throughout the experiment and the impact on mitochondrial function determined. Cancer cell lines commonly show the Warburg effect, metabolizing glucose via aerobic glycolysis under standard culture conditions (i.e. not using OXPHOS). Cells were plated (50,000 per well) and left to adhere for 6 hours before Luxcel MitoXpress Intra probe was added and cells incubated overnight (5% CO _2_, 20% O _2_). Cells were washed in PBS and media changed as follows: INS-1, 11mM glucose; HepG2, 25mM glucose; and U87MG, 25mM glucose. All treatments were in triplicate wells. Antimycin A (1µM) was used to inhibit oxygen consumption. Using the plate reader’s atmospheric control unit and with the plate lid off, the oxygen levels was reduced in a step wise manner. The conversion of fluorescent values to lifetime fluorescence values ^40^ reduces assay noise and allows the construction of an oxygen calibration curve with a particular lifetime value reflecting a specific oxygen concentration. The antimycin A treated wells, where mitochondrial respiration was inhibited, were used to construct a calibration curve so that intracellular oxygen concentrations could be quantified from lifetime fluorescence values at each oxygen concentration. Percentage intracellular oxygen concentrations were calculated from the following equation as described previously ^41^ [% O ] = A exp(-BT) where the numerical values of the parameters A = 370, B = 0.1 µs ^-1^, and T = lifetime value (µs).

### Mitochondrial respiratory control ratio (RCR) measurements

Mitochondria from MIN-6 and INS-1 cells were isolated as follows. Cells were grown to 70% confluency in 3 × 175cm ^2^ tissue culture flasks and washed with PBS prior to removal, using a cell scraper and 12 ml of ice cold isolation buffer (ISO buffer) (0.25M sucrose, 20mM Hepes, 2mM EGTA,10mM KCl and 1.5mM MgCl). To reduce cellular superoxide levels 100µM Tiron was incubated with cells in culture. Cells were lysed by shearing on ice, using a Teflon glass homogenizer driven by a motorized stirrer (Heidolph). Large debris, including un-fragmented cells, was removed by centrifugation at 1,100×g for 10 minutes at 4°C. The mitochondria were pelleted by centrifugation at 11,000×g for 10 minutes at 4°C, washed once with 5 ml of ISO buffer and centrifuged at 11,000×g for 5 minutes. Mitochondrial pellets were re-suspended in 300–500µl of ice cold oxygen consumption buffer (0.25M sucrose, 5mM MOPS, 5mM KH _2_PO _4_ and 5mM MgCl _2_). Mitochondrial protein levels were determined using a BCA Protein Determination kit (Pierce) and mitochondria introduced into the oxygen consumption chamber (Hansatech) at a final concentration of 0.5 mg/ml. De-fatted BSA (Sigma) was added to the oxygen consumption buffer to give a final concentration of 0.1%. State 4 (Substrate alone: Glycerol-3-Phosphate (G-3-P) 5mM) and state 3 (substrate + 1mM ADP) respiration rates ^42^ were determined for each mitochondrial prep, and the respiratory control rates (RCR) calculated. G-3-P was a used as a substrate, due to its high rate of mitochondria oxygen consumption in isolated INS-1 mitochondria with previous studies indicating that Glycerol-3-phosphate dehydrogenase is expressed at high levels in pancreatic β cells ^43^. G-3-P is taken up by mitochondria using the G-3-P shuttle ^44^.

### Cellular ATP assay

*Short term low glucose exposure:* INS-1 cells were plated at a density of 50,000 cells per well and were incubated overnight in normal culture media. The media was removed and cells washed twice in glucose free media. INS-1 cells were subjected to either one hour pre-incubation in media containing 1mM glucose or incubated directly in media containing 0mM, 1mM, 2.5mM, 5mM, 10mM and 20mM glucose. Cells pre-incubated in 1mM glucose were then incubated for a further one hour in the different glucose concentrations. All incubations were carried out at 37°C under 5% CO _2_. At the end of the incubation, media was removed and the ADP/ATP ratio of the cells measured using EnzyLight ^TM^ ADP/ATP Ratio Assay Kit (BioAssay Systems), according to the manufacturer’s instructions.

### Immunoblotting

Protein was extracted from INS-1 cells using a cell extraction buffer (50mM Tris-HCl (pH 7.4), 150mM NaCl, 1 mM EDTA, 1% Triton, 0.1% SDS), containing Mini Complement protease inhibitors (Roche) and phosphatase inhibitor cocktails 1 & 2 (Sigma) at 4°C. Samples were denatured in standard SDS/PAGE loading buffer and 10µg of protein run on a NuPage 4–12% Bis Tris gel in MOPS buffer (Invitrogen). Proteins were transferred onto Hybond C-extra nitrocellulose membrane (Amersham Biosciences), according to manufacturer’s instructions. Blocking was performed using 5% fat-free milk solution in 1 X TBS (0.05% Tween) for one hour at room temperature. The levels of ER stress associated proteins were analyzed using the following antibodies: alpha subunit of eukaryotic initiation factor 2 (eIF2 α; 9722) and binding immunoglobulin protein (BiP; C50B12) rabbit polyclonal antibodies (both 1:1000, Cell Signalling Technologies); rabbit SOD2 antibody (ab13534; 1:2000; Abcam); mouse monoclonal antibody to β-actin (ab8226; 1:10,000; Abcam). Specificity of antibodies has been shown previously (see manufacturer’s websites for published articles and validation) with each identifying a single band of the appropriate size on western blots. Blots and primary antibodies were incubated over night at 4°C in TBS-T containing 5% BSA. The secondary antibody HRP-conjugated rabbit anti-mouse or goat anti-rabbit antibody (Dako) was incubated for 2 hours at a 1:5000 dilution at room temperature in 1 X TBS. Chemiluminescence detection was conducted with the ECL Plus Western Blotting Detection System (Amersham Biosciences) and X-ray film, according to the manufacturer’s instructions. Quantification of bands using densitometry was carried out using image J (version 1.54).

### Measuring autophagy using Image Stream

Image Stream (IS100; Amnis) is a multispectral flow cytometer combining standard microscopy with flow cytometry. It can acquire up to 100 cells/sec, with simultaneous acquisition of six images of each cell, including brightfield, scatter, and multiple fluorescent images. We used the integrated software INSPIRE (Amnis) to run the Image Stream. Cells were removed from flasks using Accutase, as outlined previously. For each experiment, 500,000 cells were stained with Lyso-Id (Enzo Life Sciences; ENZ-51005-500) and live dead marker (Invitrogen; L34955), according to the manufacturer’s instructions. Cells were then fixed and permeabilised (ebioscience Fixation and permeabilisation Kits; 00-8222-49 and 0083333-56, respectively) and stained with antibodies to LC3 at 1:100 dilution (Epitomics; rabbit antibody; MAP1LC3A, EP1983Y). This was subsequently stained using a goat anti-rabbit antibody (IgG H+/L; Invitrogen; F-2765) conjugated to FITC at a dilution of 1:400. All antibodies were titrated for Image Stream ^45^. Cells were finally suspended in 50µl of buffer (cold PBS with 1% FCS and 0.05% sodium azide) in 0.6 ml microcentrifuge tubes. At least 10,000 cells/experimental sample and 2000 cells/single color control were acquired per sample. The .rif files generated from INSPIRE were analyzed using IDEAS 4.0.735 software (Amnis). Single color control files were used to create compensated files (.cif), followed by generation of data (.daf) files. The single cells were gated for in focus cells based on brightfield gradient root mean square (GRMS) feature (>300 GRMS). Mean bright detail similarity was measured on double positive (LC3 ^hi^ Lyso ^hi^) cells. Effects of glucose concentrations were assessed by regression analysis and paired sample t testing.

### Statistical analysis

Statistical analysis was carried out using SPSS version 25. For the sample in Figure 8, normality was assessed by visual inspection of frequency plots, and groups were compared by ANOVA. For all other comparisons, nonparametric tests were preferred (Mann-Whitney U or Kruskall-Wallis test as appropriate) or carried out by ANOVA using bootstrap modification.

## Results

### Insulinoma cell lines are sensitive to media glucose levels with reduced glucose associated with increased mitochondrial ROS production

**Figure 1.**
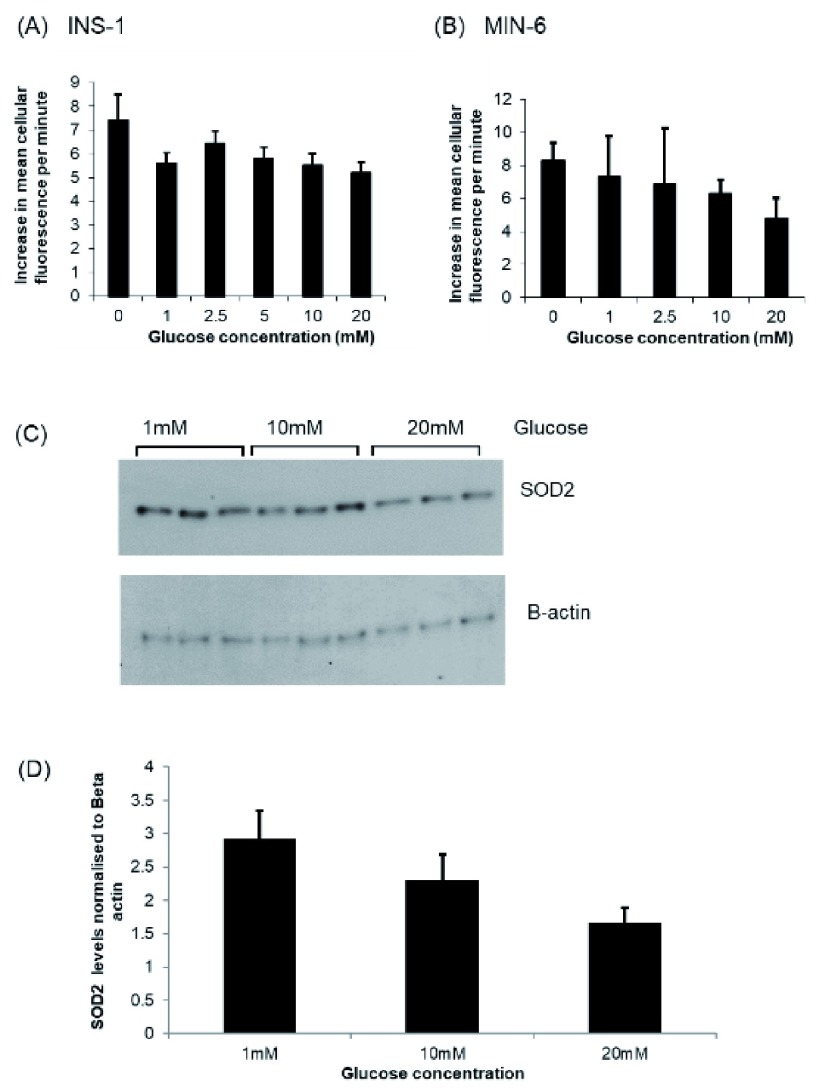
Low glucose exposure increases beta cell mitochondrial reactive oxygen species (ROS) and antioxidant levels. MIN-6 and INS-1 mitochondrial ROS production was assessed by loading cells with 5µm MitoSox followed by incubations in different glucose concentrations. ( **A**) and ( **B**) show the rate of increase in mean cellular MitoSox fluorescence per minute (Ex 488nm, Em 575+ 26nm; 10,000 cells analyzed at each time point) for INS-1 and MIN-6 cells, respectively, for two representative experiments. The log transformed rate of ROS production did not differ significantly from a normal distribution (MIN-6 n=3, INS-1 n=4). This fell significantly with increasing glucose concentration (p<0.01) for INS1 using ANOVA, but was not significant for MIN6. Levels of mitochondrial enzymes involved in controlling mitochondrial superoxide levels were assessed by immunoblotting. (C) Immunoblot showing levels of SOD2 levels (molecular weight 26.6KD runs at 24KD on the blot) and β-actin (molecular weight 42KD) following short term (90 minute) reduced glucose exposure of INS-1 cells. (D) Levels of SOD2 were determined relative to β-actin using densitometry and Image J software. Experiments were run in triplicate. Statistical comparisons to low glucose conditions (1mM) were made using unpaired t-test. The number of experiments shown in the figure are for biological replicates.

Initial experiments showed that β-cells grow poorly when media glucose levels are below 5mM ( Supplementary Figure 4A). Poor growth of MIN-6 cells in 5mM glucose under 20% O _2_ could be improved by reducing the concentration of tissue culture oxygen to 10%, implicating oxygen stress ( Supplementary Figure 4B). Further studies of the origins of the oxidative stress showed the ROS resulting from incubation in low glucose medium to be of mitochondrial origin. MitoSox analysis of ROS/mitochondrial superoxide by flow cytometry showed a significant increase in mitochondrial ROS production when cells were incubated in low glucose for 4 hours for INS-1 (p<0.001; n=4) and but not for MIN-6 (n=3) cells (calculated by ANOVA). A representative experiment for each cell line is shown in Figures 1A and B.

Three further experimental approaches suggest that this ROS increase is potentiallyis localized to mitochondria. Firstly, a tendency (not significant) to increase levels of the mitochondrial superoxide scavenger SOD2, was observed under low glucose conditions following a 90 minute glucose challenge ( Figures 1C and D). Secondly, cytoplasmic ROS levels measured by Oxyburst Green ( Supplementary Figures 5A and B) and dihydroethidium ( Supplementary Figure 4C) did not show an increase in total cellular ROS following short term low glucose exposure. Thirdly, the absence of ER stress following prolonged low glucose exposure further suggests that the oxidative stress is contained within the mitochondria ( Supplementary Figure 6A and B).

### Priming GSIS with a low glucose challenge significantly affects INS-1 insulin secretion

**Figure 2.**
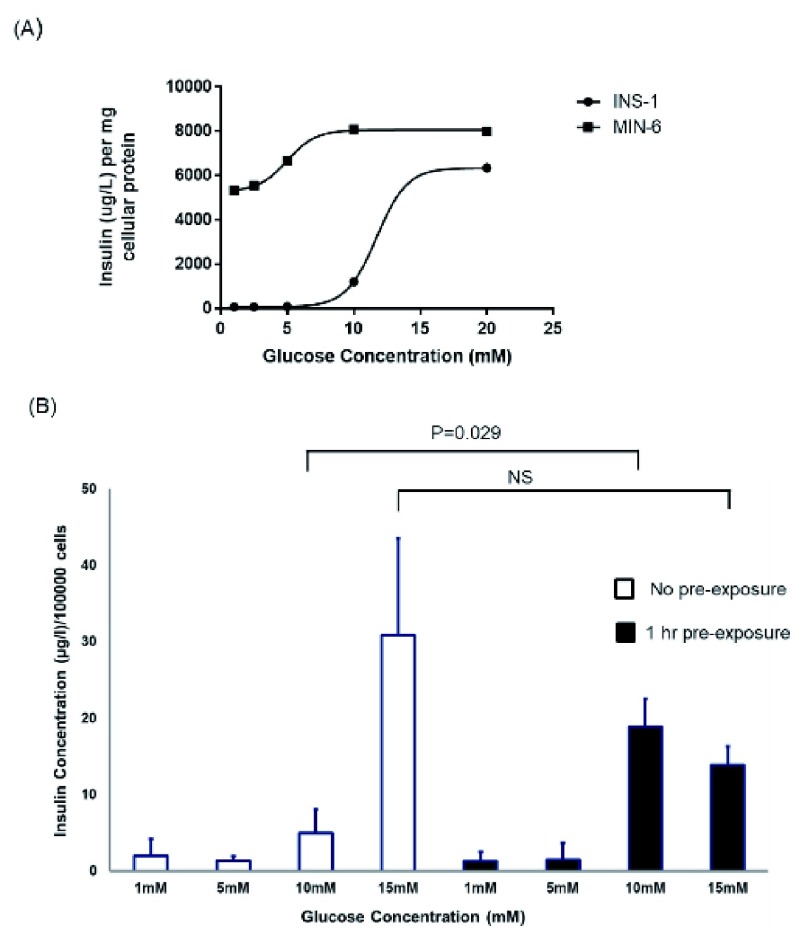
INS-1 and MIN-6 cells show a glucose stimulated insulin secretion (GSIS) response, which is altered by a pre-incubation with low glucose media. ( **A**) MIN-6 and INS-1 cells show a sigmoidal GSIS response. ( **B**) Effect of one hour 1mM glucose pre-incubation on the GSIS response of INS-1 cells (black bars) compared to cells without a pre-incubation (white bars). Data was analyzed using an unpaired t-test. Significant differences were observed when GSIS was compared between the two treatments when cells were incubated in 10mM glucose (P=0.029) (n=4 biological replicates).

In most GSIS protocols, cells are routinely exposed to low glucose prior to a glucose challenge. Because the increase in mitochondrial ROS production on reducing glucose was unexpected, we further explored the effect of low glucose challenge on GSIS. Using a standard GSIS protocol incorporating a one hour pre-incubation in 1mM glucose both MIN-6 and INS-1 showed a sigmoidal glucose stimulated insulin secretion response ( Figure 2A). The GSIS responses of the two lines differ, with the MIN-6 cells having a higher basal level of insulin secretion and more rapidly reaching maximal secretion when glucose levels are raised. Both lines secrete similar maximal levels of insulin in response to glucose. In Figure 2B, we demonstrate that subjecting INS-1 cells to a one hour pre-incubation in 1mM glucose prior to the GSIS assay affected the amount of insulin secreted. The level of insulin secreted at 10mM glucose significantly increased ( Figure 2B).

### Exposure of INS-1 cells to low glucose causes an anomalous reduction in mitochondrial respiration

**Figure 3.**
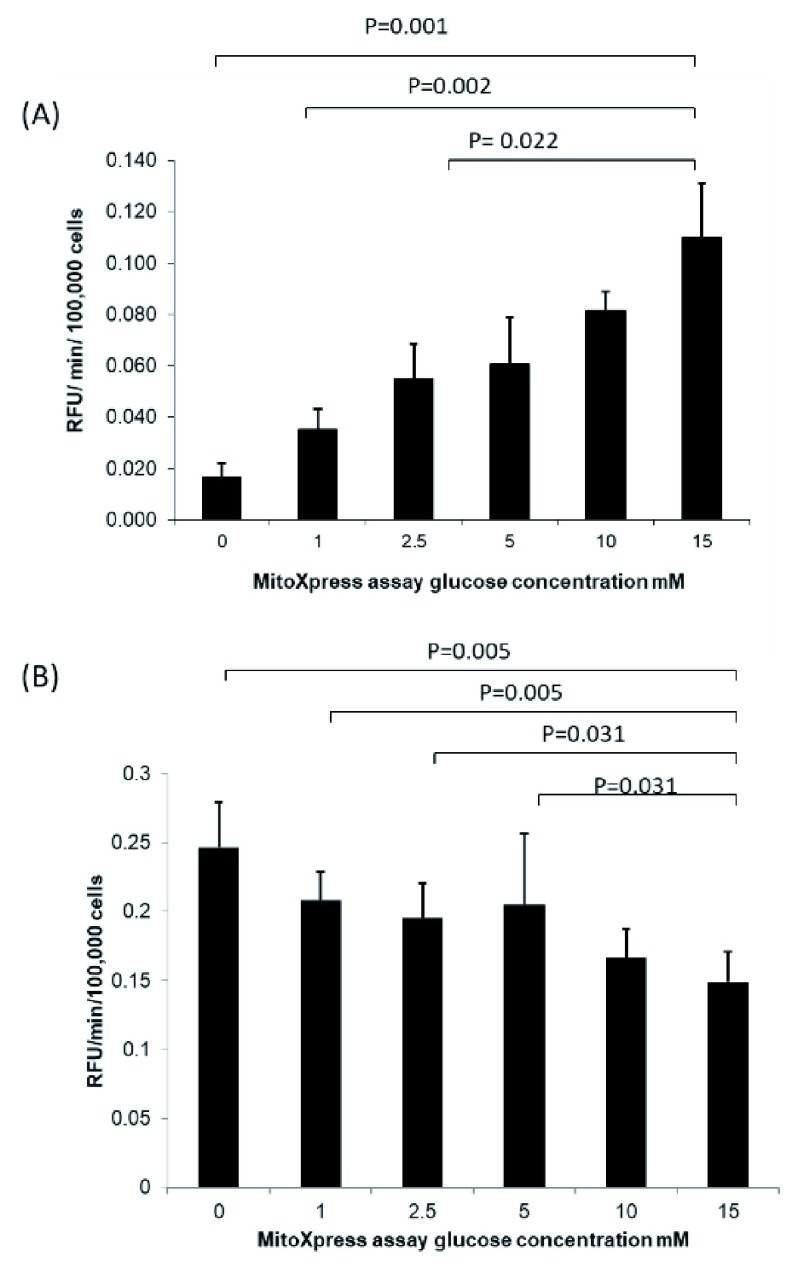
Short term reduced glucose exposure over 60 minutes reduces whole cell INS-1 respiration. Cells were grown under normal culture conditions (11mM glucose for INS-1 cells and 25mM for HepG2 cells), harvested, washed with PBS and incubated in different glucose concentrations containing the MitoXpress Xtra probe to track oxygen consumption. The assay has an initial lag phase of approximately 60 minutes before the maximal rate of oxygen consumption could be determined. ( **A**) INS-1 cells show a significant decline in oxygen consumption as glucose levels are reduced. ( **B**) This is not observed with HepG2 cells where reduced glucose exposure increases oxygen consumption and is a response to reduced glucose levels well documented in the literature ^42^. Significant differences in respiration rates of the different treatments were determined by comparing the rates to those closest to normal culture media glucose levels (10mM INS-1 and 15mM HepG2). One-way ANOVA was used for the analysis; n=18 (3 biological replicates, 6 technical repeats for each day(INS-1)) and n=6 (3 biological replicates, 2 technical repeats for each day (HepG2)).

**Figure 4.**
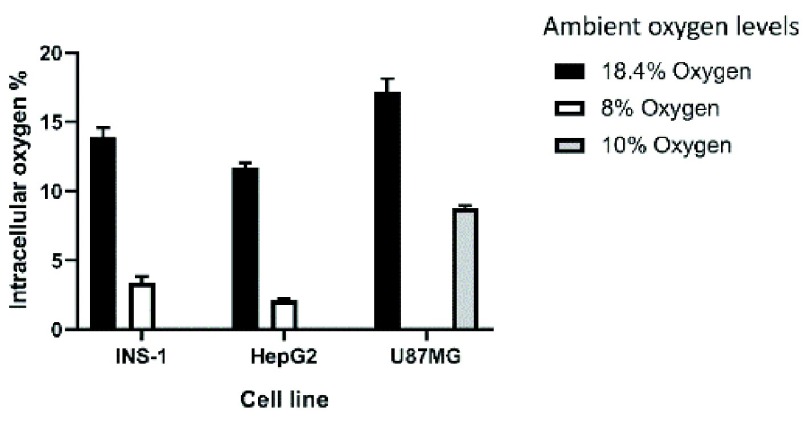
INS-1 cells show high levels of oxygen utilization under high glucose conditions. Intracellular oxygen levels were calculated using the equation [% O _2_] = A exp(-B T), where A = 370 and B = 0.1 μs ^-1^, T = lifetime value (µs) as outlined in the methods and previously described ^41^. Intracellular O _2_ levels for INS-1, HepG2 and U87MG cells were calculated when cells are exposed to ambient 18.4% O 2, 8% (INS-1 and HepG2) and 10% U87MG. For the more highly respiring INS-1 and HepG2 cell lines in high glucose media intracellular oxygen levels track below the ambient oxygen concentration in the plate reader as the cells are able to compete against oxygen diffusion into the media. The U87MG cells which have a low rate of respiration in high glucose media are unable to complete against oxygen diffusion into the well and the intracellular oxygen concentration reflects that of the environment (i.e. ambient oxygen concentration). Figure 4 comprises of 6 technical replicates from a single experiment.

When INS-1 cells were incubated in media with low glucose concentrations, whole cell oxygen consumption rapidly falls ( Figure 3A). This contrasts with other cell lines, where reducing glucose levels forces cells to use mitochondrial OXPHOS substrates, such as glutamine, increasing oxygen consumption ^46^ ( Figure 3B). The fact that INS-1 cells exhibit a high level of mitochondrial respiration, even when cultured on media containing high concentrations of glucose (10–15mM), is unusual for cultured cells ^47– 50^. INS-1 cells appear to have a high requirement for glucose to maintain viability, as they show significantly reduced growth rates and are unable to switch to other substrates, such as pyruvate and glutamine, when glucose levels are reduced ( Supplementary Figure 4). This elevated rate of oxygen consumption of INS-1 cells cultured on high glucose media is shown in Figure 4. Intracellular oxygen levels were calculated using an intracellular oxygen probe (MitoXpress, Luxcel Biosciences) and indicate high rates of oxygen consumption occurring in INS-1 cells under a range of atmospheric oxygen concentrations ( Figure 4). This contrasts with the more glycolytic U87MG cell line, which consumes very little oxygen under high glucose conditions ( Figure 4). Other lines, such as HepG2, also have high respiration rates in high glucose media ( Figure 4), but withstand reduced glucose conditions much better, presumably because they can further increase respiration by using other substrates ( Figure 3B).

### ATP levels do not increase in INS-1 cells following a high glucose challenge

**Figure 5.**
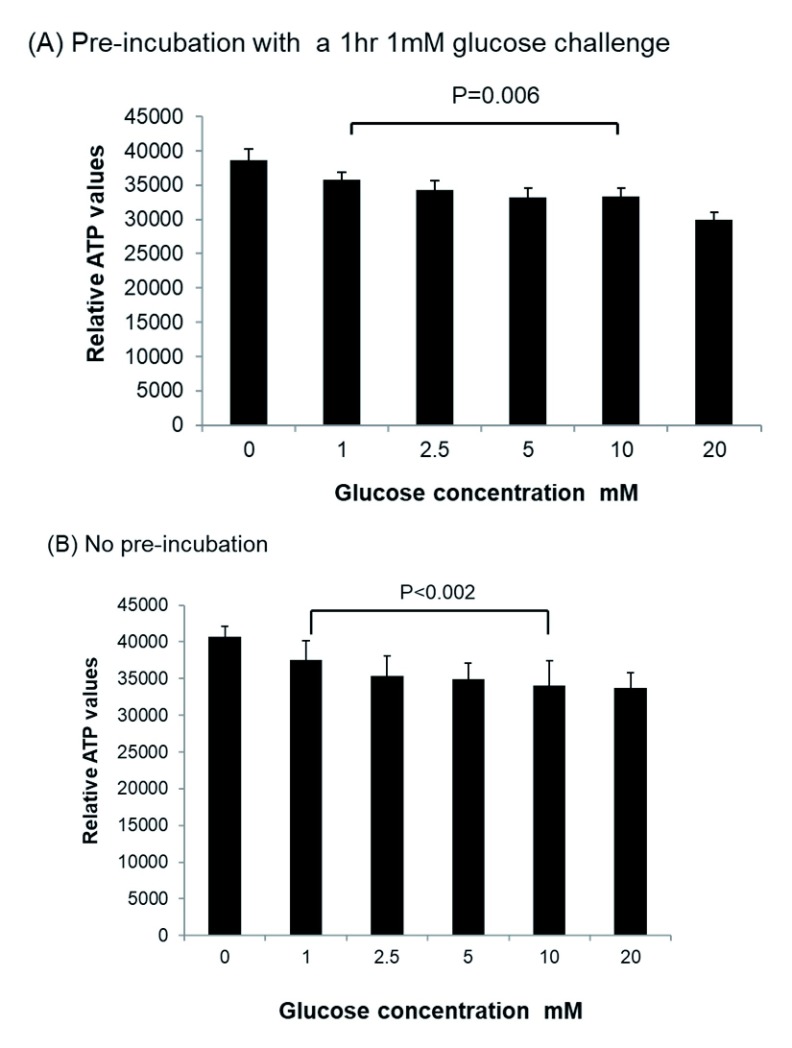
Steady state ATP levels are increased following a low glucose challenge. Steady state ATP levels in INS-1 cells following a glucose challenge. ( **A**) INS-1 cells are subjected to a 1hr pre-incubation in 1mM glucose prior to glucose exposure. ( **B**) INS-1 cells are incubated directly in the final concentrations of glucose without pre-incubation in 1mM glucose. Statistical analysis was carried out using ANOVA on three independent biological experiments. Values were compared to the 10mM glucose samples, as this is closest to normal culture RPMI media glucose levels.

In pancreatic β-cells, high glucose exposure increases intracellular levels of ATP which drives the insulin secretion response ^17, 51, 52^. However, in the INS-1 cell line increased levels of insulin secretion and respiration in high glucose media did not correlate with a rise in steady state ATP levels ( Figure 5) even though levels of respiration were elevated ( Figure 3). ATP levels were actually significantly higher in low (1mM) glucose media compared to 10mM glucose with and without a pre-1 hour low glucose exposure and subsequent glucose challenge ( Figure 5). INS-1 cells have very high levels of ATP compared to ADP under all glucose conditions testing making it difficult to observe any meaningful change in the ADP/ATP ratio. ( Supplementary Figure 7)

### Mitochondria in INS-1 cells following prolonged low glucose exposure show reduced metabolic activity, reduced mitochondrial function, whole cell respiration, and down regulated autophagy

**Figure 6.**
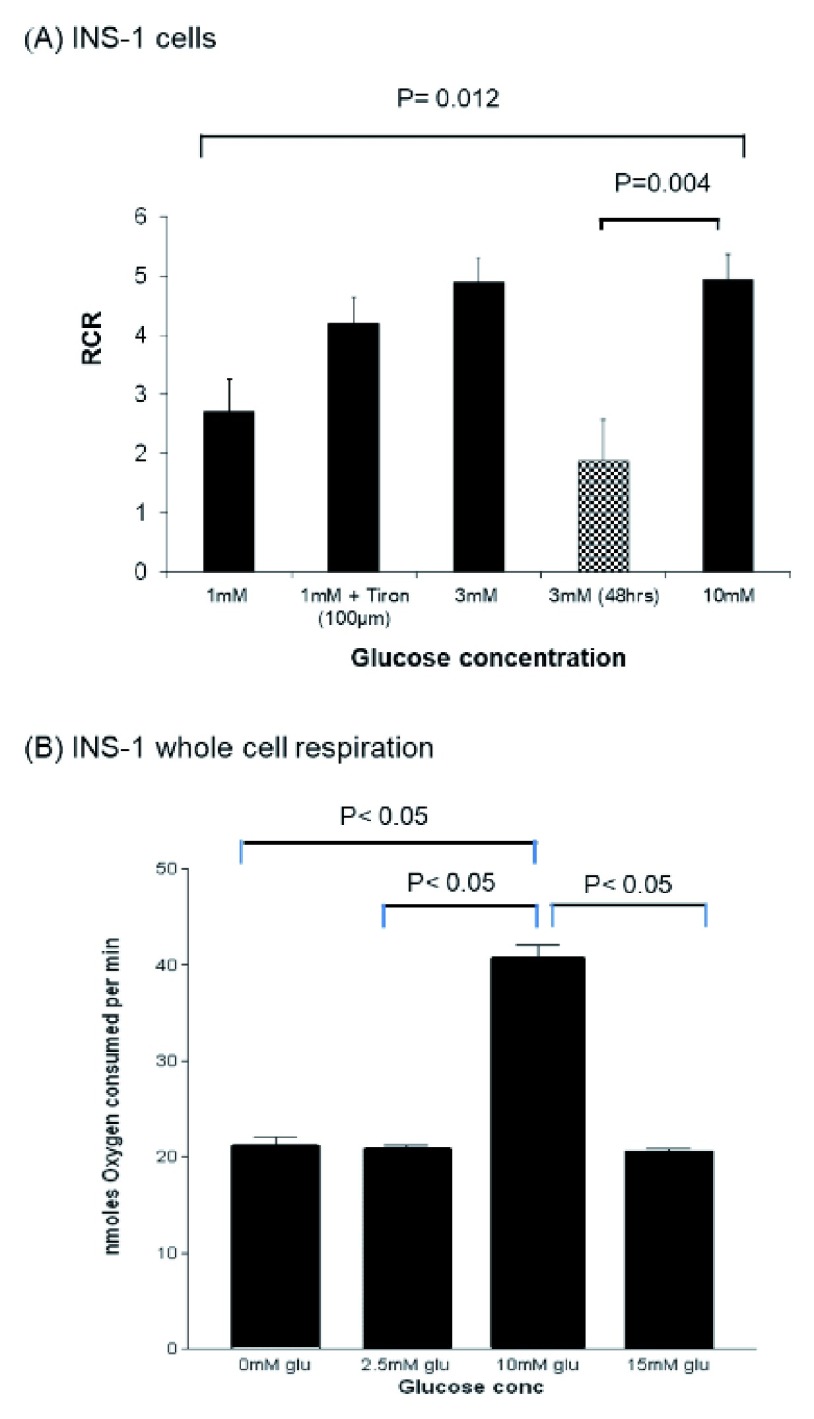
Effect of low glucose exposure on INS-1 mitochondrial function. ( **A**) INS-1 cells were incubated for 16 hours in 1mM glucose, 1mM glucose + 100µM Tiron and 3mM and 10mM glucose. 48 hour exposure was also carried out on INS-1 cells incubated in 3mM glucose (checked bar). Isolated mitochondrial respiratory control ratios (RCRs) were compared for each treatment (n=3). ( **B**) Whole cell respiration in INS-1 cells (4 million) using an oxygen electrode following 16 hour exposure to different levels of glucose (n=3). Mean cell respiration rates are compared to those obtained under optimal culture glucose conditions (10mM glucose). Data were analysed using a Mann whitney test. Both data sets were from 3 biological replicates.

**Figure 7.**
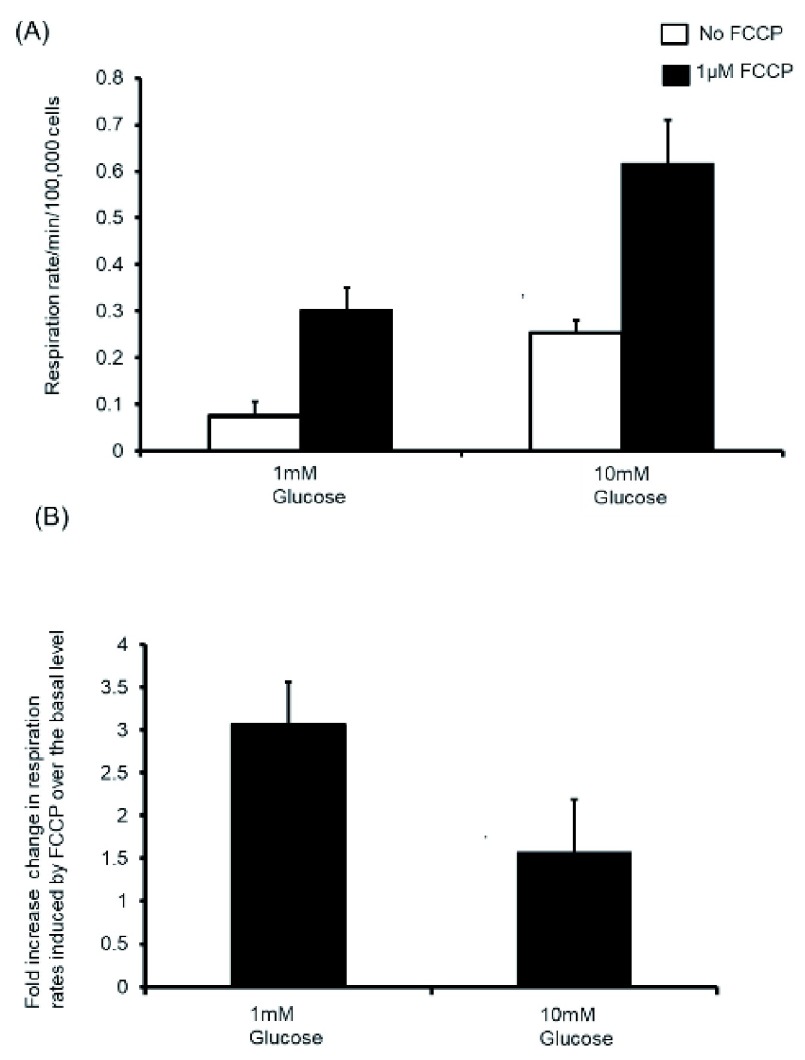
Exposure to reduced glucose conditions increases the spare respiratory capacity. To determine whether the increased reactive oxygen species (ROS) production and reduced whole cell respiration mitochondrial dysfunction could be linked to changes in the control of OXPHOS, we determined whether mitochondria demonstrated altered spare respiratory capacity. Whole cell respiration rate of INS-1 cells incubated for 16 hours in 1mM and 10mM glucose was determined in the presence of the mitochondrial uncoupler FCCP. A range of FCCP concentrations were used with 1µM giving the maximum increase in whole cell respiration rate for both 1mM and 10mM treated samples ( Supplementary File 1). ( **A**) A representative experiment with whole cell oxygen consumption rates determined for 1mM and 10mM glucose incubated samples with and without 1µM FCCP included in the assay. ( **B**) The results of three independent experiments with an increase in whole cell respiration rates due to FCCP addition relative to the basal rate. The 1mM glucose samples showed a significantly higher spare respiratory capacity (p<0.05, unpaired paired t-test) than cells incubated in 10mM glucose. Statistical analysis was carried out by ANOVA method with bootstrap modification (interaction of glucose concentration with presence of FCCP).

Isolated mitochondria from the INS-1 cells that were subjected to 16 hours incubation in medium containing 1mM glucose showed a significantly reduced mitochondrial respiratory control ratio (RCR; which was decreased to 2.7 ± 0.6 from 4.9 ± 0.2 in 10mM glucose, P<0.01; Figure 6A). This reduction could be rescued by ROS acceptors with the RCR increasing from 2.7 ± 0.6 to 4.2 ± 0.4 in 1mM glucose media on addition of 100 µM Tiron ( Figure 6A). In 3mM glucose, INS-1 cells required a longer (48 hour) incubation to initiate a significant reduction in mitochondrial RCR levels to 1.88 ± 0.7 (P<0.01; Figure 6A). MIN-6 cells incubated in 1mM glucose showed a similar trend, but this did not reach significance ( Supplementary Figure 7). The RCR values for mitochondria prepared from cells growing on low glucose were consistently lower than for cells growing in standard tissue culture glucose concentrations (11mM glucose for INS-1 and 25mM for MIN-6), ( Figure 6A and Supplementary Figure 8). These results were supported by measurements of whole cell respiration where cells were incubated for 16 hours in the different substrates ( Figure 6B). This differs from experiments in Figure 3A where we determined the immediate effects of switching to different concentrations of glucose on mitochondrial respiration. Whole cell respiration in INS-1 cells is primarily due to mitochondrial respiration because it can be almost completely blocked by the mitochondrial complex II inhibitor antimycin A. This is shown in Figure 4, when treated with antimycin A, intracellular oxygen levels in INS-1 cells reflect atmospheric oxygen. After 16 hours exposure to 1mM and 2.5mM glucose, culture glucose levels were not significantly depleted in INS-1 cells, yet the cells cultured under high glucose depleted the media more rapidly ( Table 1). This unexpected result was reflected in the media lactate levels with cells on the high glucose media producing lactate at a faster rate ( Table 1). Accumulation of lactate likely reflects the low activity of lactate dehydrogenase A in INS-1 cells ^43, 53^. In addition, long term experiments over 16hrs show that although total respiration was impaired under low glucose conditions ( Figure 7A), mitochondria under low glucose conditions showed higher rate of maximal mitochondrial respiration (FCCP treatment) relative to the basal respiration rate ( Figure 7B; p<0.05) compared to cells on high glucose conditions.

**Table 1.** Media glucose and lactate levels following a 16 hour incubation of INS-1 cells under different glucose concentrations. (Base line media lactate concentration at time 0=1mM; n=3).

Mean			Mean		
Sample numbers	Glucose added to media	Media glucose concentration mM 16hrs	STD	Media lactate concentration mM 16hrs	STD
1,6,11	1mM	1.5	0	1.1	0.21
2,7,12	2.5mM	2.6	0.15	1.1	0.17
3,8,13	5mM	4.5	0.41	1.6	0.15
4,9,14	10mM	8.2	0.64	2.4	0.55
5,10,15	15 mM	12.3	1.39	2.8	0.71

**Figure 8.**
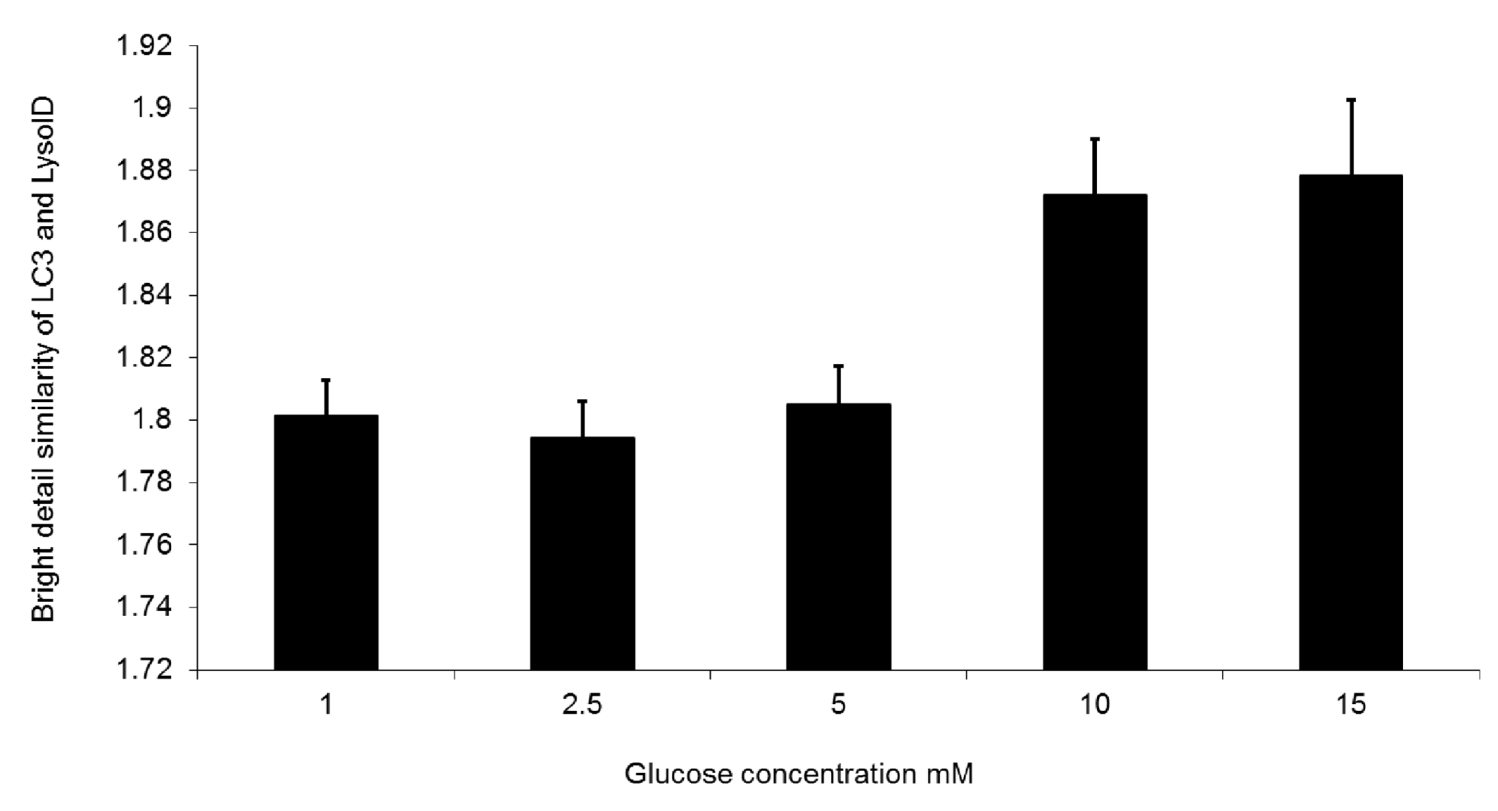
INS-1 cells cultured under reduced glucose conditions show reduced levels of autophagy. INS-1 cells were cultured in different concentrations of glucose (1, 2.5, 5, 10 and 15mM) for 16 hours and stained for anti-LC3 (autophagosome marker) and Lyso-Id (lysosome marker). The cells were run on Image stream, and 100,000 cells were acquired/treatment. Bright detail intensity was measured on double positive (LC3 ^hi^ Lyso ^hi^) single in focus live cells. Exposure to reduced glucose significantly decreased the bright detail similarity of co-localizing LC3 and the lysosomal marker, Lyso-ID (p<0.02). This was reproducible: bright detail similarity being reduced in cells incubated in 1mM compared to those in 10mM glucose respectively over three runs (unpaired test; p=0.02).

We anticipated that prolonged exposure to reduced glucose would increase autophagy both as a starvation response and for removal of damaged mitochondria. Initial studies using a standard western blot to determine the levels of the activated form of LC3 (which is linked with autophagy) showed INS-1 cells have very high levels of the lipidated LC3 (LC3II) under all glucose conditions ( Supplementary Figure 9). Image Stream combines flow cytometry and high-resolution fluorescence microscopy in a single platform, and enables detection of autophagosomes as LC3 punctae co-localizing with lysosomes using bright detail similarity. This method has been validated for detecting autophagy ^45, 54^. Following incubation we found a significant decrease in the bright detail similarity of co-localizing LC3 and the lysosomal marker, Lyso-ID (autophagy) at low glucose (p<0.02; Figure 8). This was reproducible, bright detail similarity being reduced in cells incubated in 1mM compared to those in 10mM glucose respectively over three runs (paired sample t-test P<0.02). We used HepG2 cells treated with lysosomal inhibitors in starvation medium (HBSS) as a positive control for increased co-localization ( Supplementary File 1).

## Discussion

In this study, we induce energetic stress in two insulinoma cell lines, INS-1 and MIN-6 to determine whether they are suitable models for studying the effects of mitochondrial dysfunction *in vitro*. Initial studies showed that INS-1 cells have a high rate of mitochondrial oxygen consumption in normal culture media (11–15mM glucose) compared to other cancer derived cell lines, which tend to rely more heavily on glycolysis. High levels of autophagy have recently been associated with high levels of mitochondrial respiration and are shown to be essential for the turnover of damaged mitochondria and maintenance of high ATP levels ^55^. Here we show that INS-1 cells have very high levels of autophagy, indicated by high levels of the lipidated autophagy marker LC3-II, further supporting their reliance on OXPHOS or indicating a block in the autophagy pathway. Both insulinoma lines showed a GSIS response and are sensitive to moderate reductions in media glucose (< 5mM); reduced glucose not only resulted in poor growth, but also increased levels of oxidative stress. Unlike previous work showing that oxidative stress caused by high levels of macronutrients (glucose, lipids) and pro-inflammatory cytokines impair β-cell viability ^56, 57^, the oxidative stress that we documented appears to be confined to the mitochondria. Furthermore, this did not initiate long term ER stress. Within two hours of low glucose exposure, levels of mitochondrial superoxide and superoxide scavenger SOD2 increased. Exposure to high glucose concentrations rapidly elevated mitochondrial respiration, but this did not correlate with an increase in cellular ATP levels, even though an increase in insulin secretion was observed. Prolonged exposure to reduced glucose conditions significantly impaired mitochondrial function and reduced levels of autophagy.

By relying on mitochondrial respiration to trigger the GSIS response, insulinoma lines reflect primary β-cells, which are very sensitive to perturbations of mitochondria function by mtDNA mutations or inhibitors. Previous studies by Sekine *et al.* ^43^ showed that glycolysis and mitochondrial oxidation are closely coupled in INS-1 cells. Others have linked the GSIS profile ^58^ and resulting β-cells viability ^59^ to glucokinase activity. Our data also indicates that the GSIS profile is affected by glucose conditions immediately before the glucose challenge. A pre-incubation in 1mM glucose increases the levels of insulin generated under moderate glucose conditions (10mM), but reduces insulin secretion under high glucose conditions (15mM). Previous studies have shown that ROS can trigger insulin secretion, Hence, further studies are required to determine whether the high mitochondrial ROS or the variation in low glucose concentrations is the major priming event for subsequent GSIS. As glucose conditions are constantly fluctuating *in vivo*, it is possible that the β-cell response to low glucose is used to prime the insulin secretion response when glucose levels are raised. While mitochondrial uncoupling is already known to modulate insulin secretion ^24^, the potential role of ROS in priming of the β-cell to respond to a subsequent glucose challenge is a novel finding. The link between an increase in mitochondrial ROS and increased insulin secretion is supported in studies that show that increased mitochondrial ROS production can increase insulin secretion ^18, 24^.

ROS increases did not result in an ER stress response. Indeed, the converse was true with eIF2-alpha phosphorylation increasing in high glucose media. Cancer cell lines in general show reduced levels of mitochondrial respiration following prolonged incubation in media containing high concentrations of glucose ^27, 60^. Reducing glucose concentrations <1mM drives the cancer cells to use other energy substrates, such as glutamine and fat, to fuel mitochondrial respiration ^39, 46, 61^. In contrast, insulinoma INS-1 cells do not increase OXPHOS in response to low glucose ( Figure 3). Furthermore, exposure of INS-1 cells to 15mM glucose results in an immediate stimulation of mitochondrial respiration which is lost by 16 hours ( Figure 6 (B)). Given that INS-1 cells are normally grown in RPMI which has a glucose concentration of 11mM, it is likely that prolonged exposure to 15mM glucose is deleterious to INS-1 cells and hence mitochondrial respiration falls. Moreover, *in vitro* experiments using β-cell lines and isolated pancreatic islets are generally carried out under non-physiological oxygen conditions (20%) which could impact on mitochondrial function and ROS production ^62, 63^. Further studies of other beta cell models including bHC, BRIN-BD11, Blox5 which show a strong GSIS response are needed to determine whether our findings reflect general properties of beta cells that are required for their physiological role. An intriguing finding with INS-1 cells is that glucose stimulation did not result in an increase in steady state ATP levels or the ATP/ADP ratio, whether or not there was a one hour pre-incubation in 1mM glucose. Similar results were found by Sarre and colleagues ^64^, who also showed that ATP levels were not elevated during GSIS assay. As steady state levels of ATP do not appear to rise in INS-1 cells following glucose stimulation, it is possible that additional mitochondrial secretalogues are generated by increases in mitochondria respiration ^14^ and is sufficient to drive the increases in insulin secretion in INS-1 cells rather than a global increase in cellular ATP. Increases in ATP could be more localized to the sites of insulin release with no significant changes seen at the cellular level as cell anabolism increases to produce more cellular constituents ready to proliferate. Such changes would not be observed using the techniques employed in this study.

ATP/ADP ratios proved difficult to interpret due to the very low levels of ADP detectable in the INS-1 cell line. The fact that ATP levels remain high in β cells exposed to low glucose with little glucose utilization suggests that INS-1 cells have a mechanism that can conserve ATP levels or generate ATP more efficiently. This could be simply linked to reduced cellular energy demands under low glucose conditions (i.e. minimal insulin secretion), which may account for the observed net rise in cellular ATP. Alternatively, the mitochondria may be better coupled in low glucose. Interestingly, knockout of UCP2 in β cells increases the amount of insulin secreted under high glucose conditions, highlighting the need for controlling mitochondrial energy production even when the β cell is operating under conditions of high energy need ^24^.

In conclusion, our data suggests that insulinoma cell lines show a clear requirement for glucose driven mitochondrial OXPHOS linked to insulin secretion. This is unlike most cancer lines, where glucose suppresses mitochondrial OXPHOS and causes the cells to rely on glycolysis for the generation of ATP and cell growth. Inducing energetic stress by reduced glucose exposure resulted in mitochondrial dysfunction and reduced autophagy over 24 hours, which was associated with increased ROS production. In the short term, pre-incubation with low glucose modulated the insulin secretion response, inducing cells to respond to more physiological levels of glucose at 10mM. This data makes insulinoma cell lines very attractive models to investigate perturbations of mitochondrial function by knocking out genes that cause mitochondrial diseases. Compounds, including Zidovudine, that reduce the levels of mitochondrial DNA could also be studied to investigate the mitochondrial mutation threshold effects observed in mitochondrial disease patients *in vivo*. The lack of an increase in ATP or the ATP/ADP ratio upon glucose stimulation is in contrast with the canonical increase in ATP or ATP/ADP ratio following a glucose challenge to β-cells. As cancer cells, INS-1 cells are likely to have an improved stress survival response compared to primary cells, and this may explain their ability to withstand long term substrate withdrawal and enter a metabolically quiescent state, which maintains levels of cellular ATP, without triggering ER stress or increasing autophagy.

## Data availability

Raw data for all figures, as either excel spread sheets, prism files or uncropped immunoblots, are available at figshare: doi: 10.6084/m9.figshare.5395453

## Supplementary files

**Supplementary Figure 1. Optimisation of mitoSox concentrations to monitor mitochondrial ROS production using flow cytometry.** A ten minute incubation with 5µm mitosox produced a linear increase in mitochondrial ROS when cells were incubated under ROS inducing low glucose conditions. Panels ( **A**) and ( **B**) show the effect of incubating MIN-6 and INS-1 cells with differing concentrations of mitoSox for 10 minutes prior to ROS induction with 0mM glucose. 0mM glucose was used as this gives the max induction of mitochondrial ROS in the INS-1 and MIN-6 cell lines. The mean cellular fluorescent values were the mean of 10,000 cells counted by flow cytometry. Incubation with 1µM or 0.5µM mitoSox for 10 minutes failed to detect the increases in superoxide above background levels.

**Supplementary Figure 2. Example of how whole cell respiration rates are calculated using MitoXpress xtra.** An example of the raw data used to calculate the relative rates of oxygen consumption (MitoXpress assay) when INS-1 cells are exposed to low glucose is shown above. Rates of oxygen consumption are determined in the linear portion of the assay (blue box) when the rate of oxygen consumption is at its maximum.

**Supplementary Figure 3. A typical intracellular O _2_ experiment using HT1080 cells and MitoXpress Intra.** A typical intracellular O _2_ experiment using HT1080 cells and MitoXpress Intra. The probe was loaded into the cells in a 96 well black plate overnight, cells washed and antimycin A added to some of the wells to inhibit mitochondrial respiration. Intracellular O _2_ levels are then monitored in real time in a fluorescent plate reader. Fluorescence values are converted to intracellular O _2_ levels using a calibration curve and equation. Modulation of O _2_ levels in the plate reader allows intracellular O _2_ levels to be measured over a range of ambient O _2_ concentrations. In HT1080 cells with high levels of mitochondrial respiration intracellular O2 levels in the cells are consistently lower than the atmospheric O _2_ levels and the levels in the antimycin A treated cells.

**Supplementary Figure 4. The effect of increasing glucose and oxygen concentration on cell growth characteristics.** Growth of MIN6 (squares) and INS-1 cells (triangles) over 3 days in media containing 0–20mM glucose ( **A**). Tissue culture oxygen was 20% and CO _2_ 5%. Flasks were inoculated with 1 × 10 ^6^ and 2 × 10 ^6^ of MIN-6 and INS-1 cells, respectively. Each data point is the mean of three separate experiments. Panel ( **B**) shows the effect of reduced tissue culture oxygen concentrations on the growth of MIN-6 cells grown in 5mM glucose, 5% CO _2_ over an 8 day period. Three separate flasks were used for each oxygen concentration. Significance was assessed using Student’s t–test.

**Supplementary Figure 5. Whole cell peroxide and superoxide production under reduced glucose conditions.** For panels ( **A**) & ( **B**) MIN-6 and INS-1 cells are shown in black and grey bars, respectively. Cells were harvested, washed in PBS and labelled with DCFDA, SE (whole cell peroxide) and incubated in different glucose concentrations as outlined in Methods. The data shown is for one hour incubation. Each data point is the mean of three independent experiments (10,000 events counted for each) and the data expressed as mean fluorescence intensity per cell. In panel ( **C**), whole INS-1 cell superoxide levels following reduced glucose exposure were assessed by monitoring levels of 2-hydroxyethidium. Cells were labelled with DHE for 20mins in normal culture media (RPMI 11mM glucose), washed and then incubated in different concentrations of glucose in RPMI media for 90mins. Experiments were carried out in triplicate and no significant effects were observed.

**Supplementary Figure 6. Long term reduced glucose exposure does not induce a stress response in INS-1 cells.** Levels and activation of ER stress proteins were determined by immunoblotting whole cell protein extracts from INS-1 cells grown for 24 hours under different glucose concentrations. ( **A**) Levels of BiP and eIF2-α are shown. A comparison with an antibody specific for the phos Ser51 eIF2-α indicated that the higher molecular weight band (*) is the same size as the phosphorylated form of the eIF2-α protein ( **B**) Shows the effect of the ER stress inducing compound tunicamycin (2µg/ml) on INS-1 BiP levels following 20 hour exposure in 10mM glucose with and without 50µM mecaptoethanol. ( **C**) shows quantification of the relative levels of the phosphorylated (*) eIF2-α relative to the un-phosphorylated form in INS-1 cells following 24 hours exposure to 1mM, 3mM and 10mM glucose.

**Supplementary Figure 7. A typical experiment showing changes in ATP and ADP values following a 1hr incubation of INS-1 cells in different glucose concentrations.** The figure shows the very low ADP values under all conditions tested resulting in difficulties in determining the ADP/ATP ratio. NB: Values calculated for the same well.

**Supplementary Figure 8. MIN-6 cells also show a trend towards reduced mitochondrial function on low glucose media.** MIN-6 cells were incubated for 24 hours in 1mM glucose, 1mM glucose + 100µM Tiron and 10mM glucose. Isolated mitochondrial respiratory control ratios (RCRs) were compared for each treatment. Each treatment shows the mean of three independent experiments with statistical comparisons made using Student’s t-test.

**Supplementary Figure 9. INS-1 cells express high levels of the lipidated form of LC3 associated with the autophagsome.** This makes it very difficult to quantify changes in levels of the lipidated form (LC3-II) relative to the non-autophagosome form (LC3-I) by immunoblotting.

**Supplementary File 1. Amino acid starvation increases autophagy using ImageStream in HepG2 cells.**

**Supplementary File 2. FCCP optimization as a mitochondrial uncoupling agent in INS-1 cells.**
