# Supplementary figures and images for: Insights into pancreatic β cell energy metabolism using rodent β cell models
Source: Wellcome Open Res. 2019 Sep 25;2:14. Originally published 2017 Feb 24. [Version 3] doi: 10.12688/wellcomeopenres.10535.3 (PMC6854877; doi:10.12688/wellcomeopenres.10535.3)

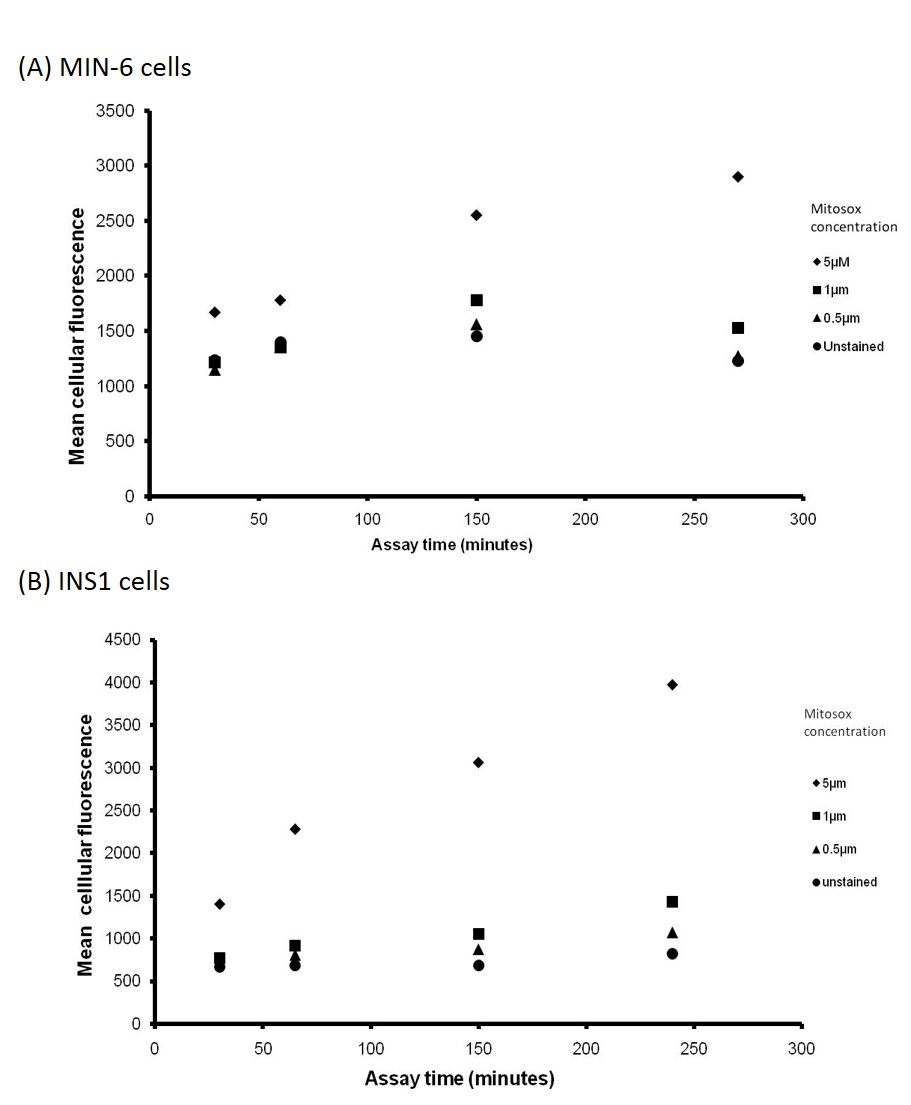

Supplement: Supplementary file 1 [file wellcomeopenres-2-16930-s0000.tgz › e91e290a-23bc-4481-b3d4-aad7a35724a1.jpg]

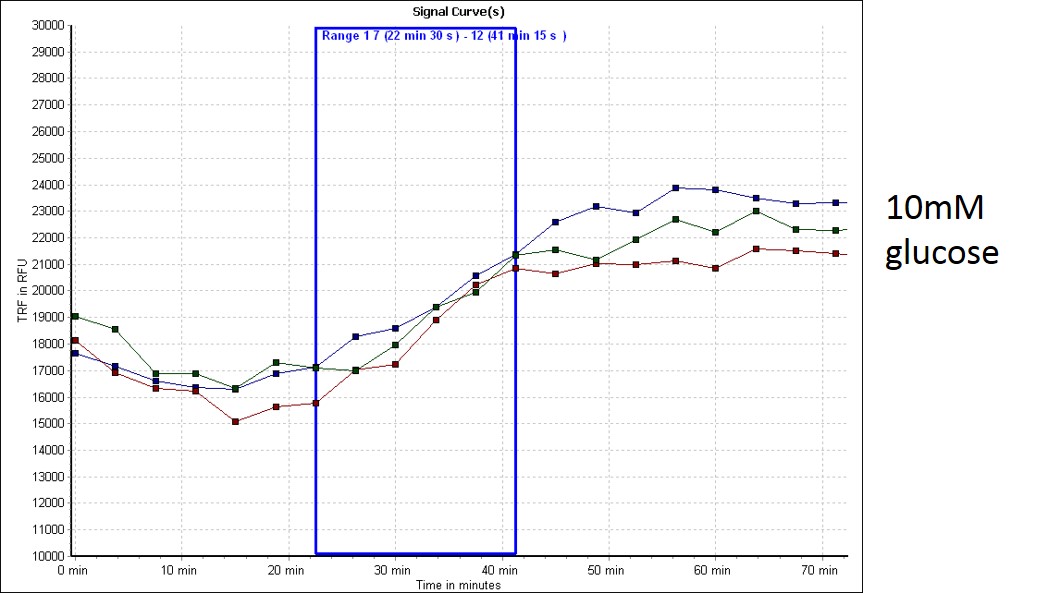

Supplement: Supplementary file 2 [file wellcomeopenres-2-16930-s0001.tgz › abe0db38-027d-4f66-b042-bfea040630d7.jpg]

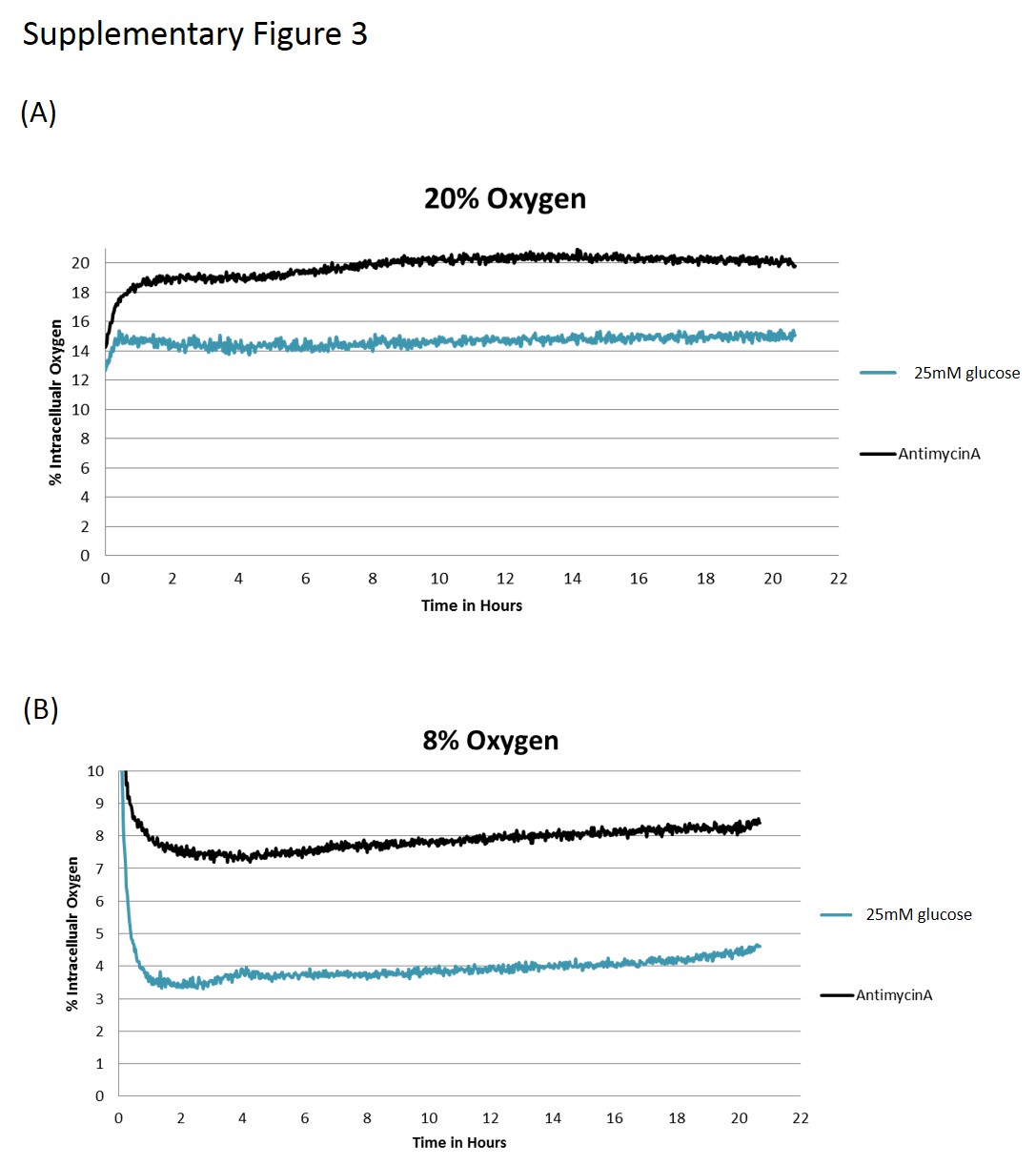

Supplement: Supplementary file 3 [file wellcomeopenres-2-16930-s0002.tgz › 448a1c10-e982-48f6-8ce6-c8e9c8837b5c.jpg]

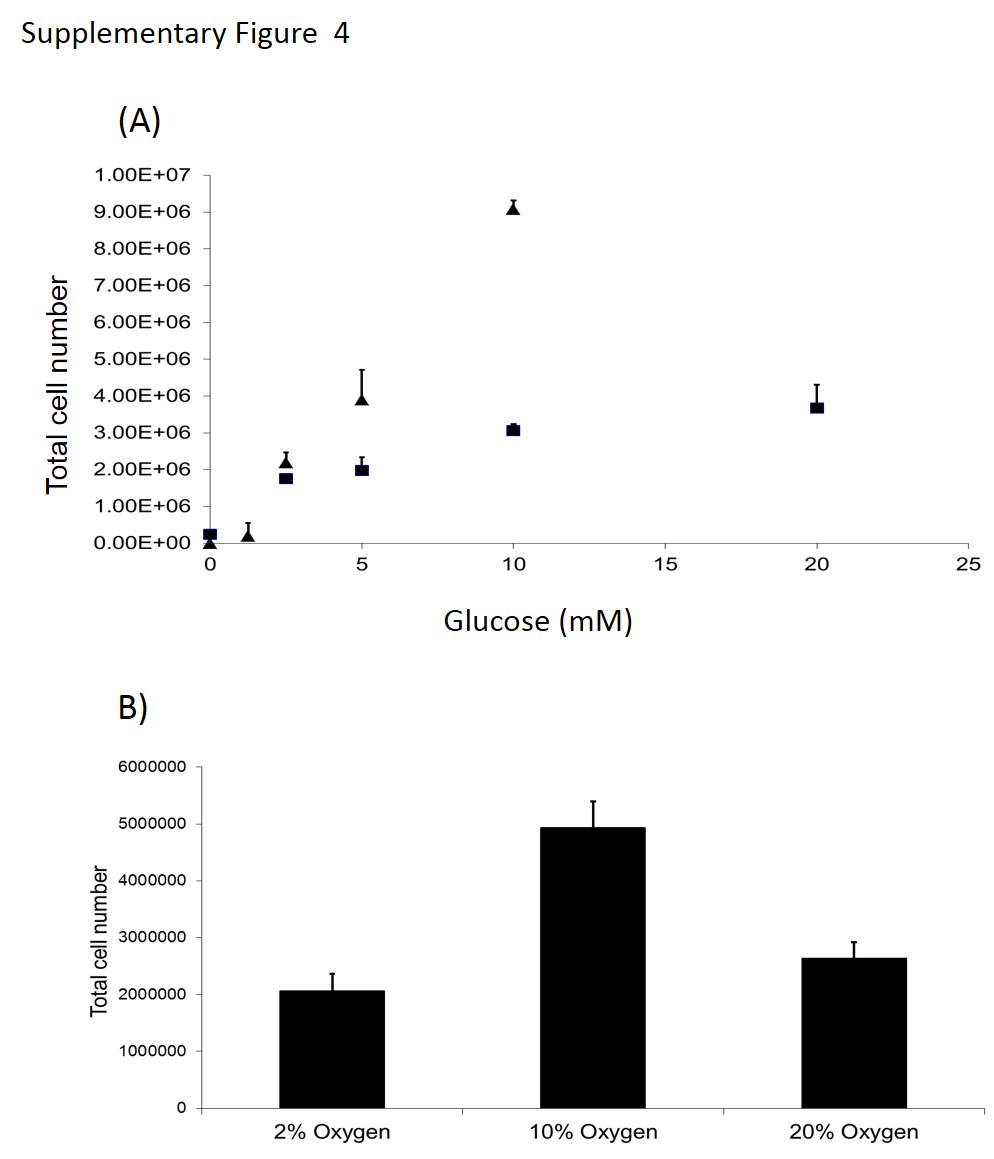

Supplement: Supplementary file 4 [file wellcomeopenres-2-16930-s0003.tgz › 8e63fb72-8d46-45b9-b6ef-8cbcd9ede0ad.jpg]

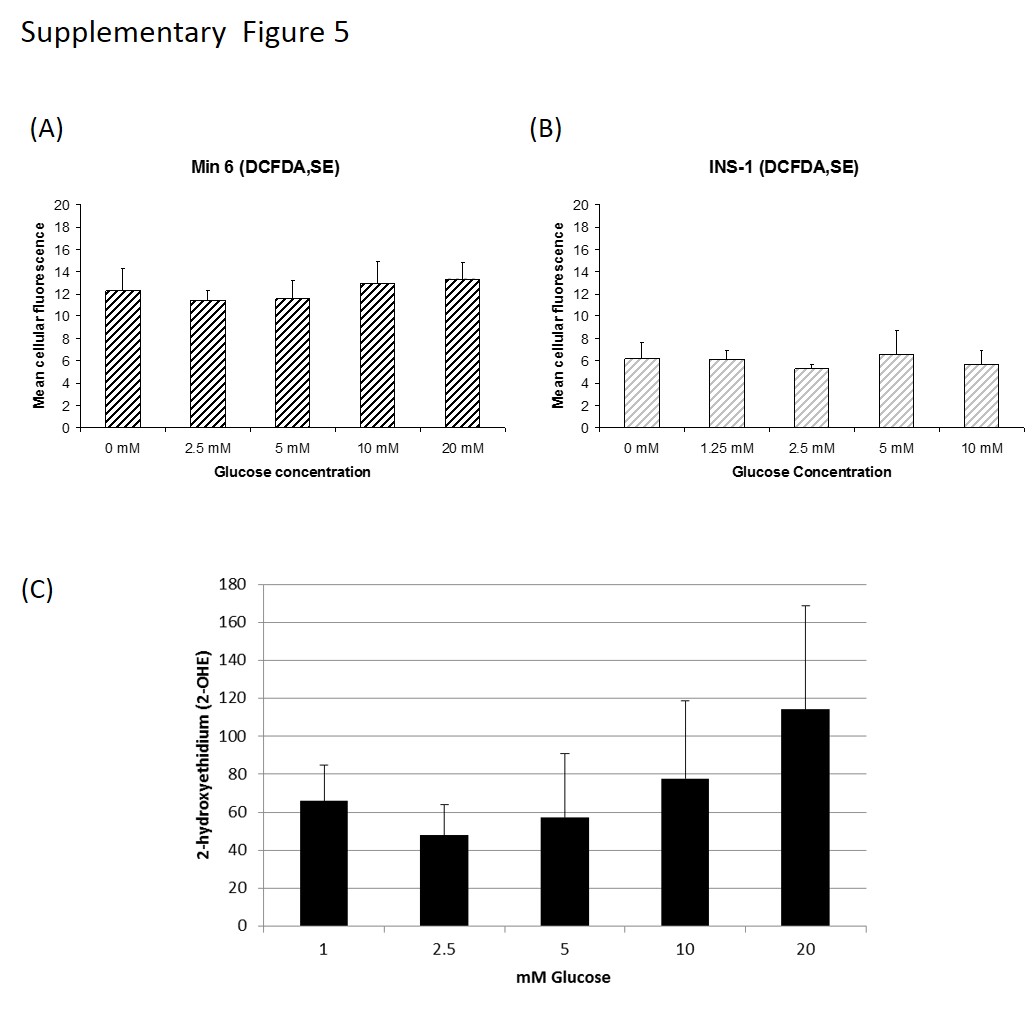

Supplement: Supplementary file 5 [file wellcomeopenres-2-16930-s0004.tgz › 850594e4-bbc7-438d-bcb2-38573dadfc29.jpg]

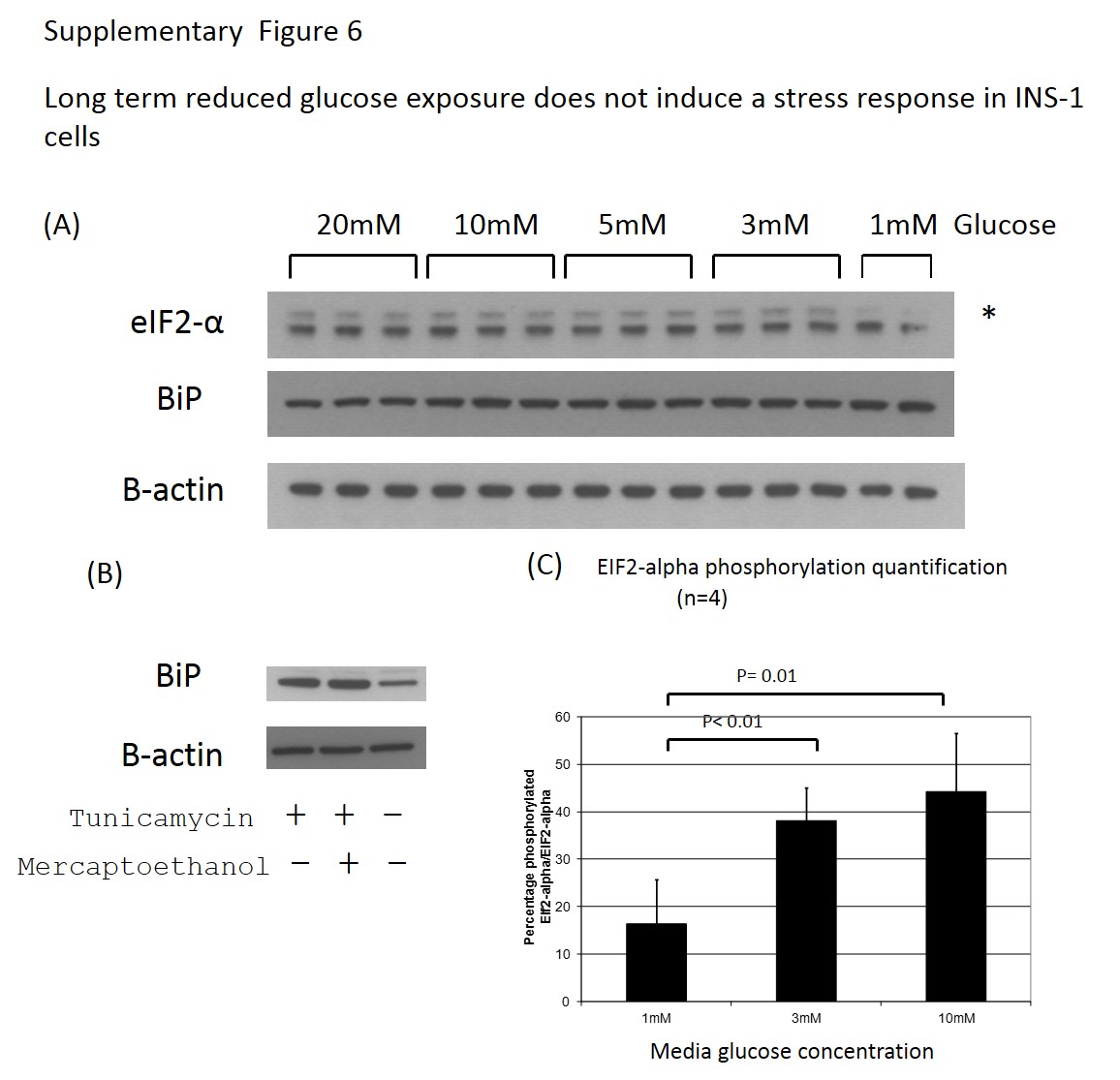

Supplement: Supplementary file 6 [file wellcomeopenres-2-16930-s0005.tgz › f401f0e8-2b40-415a-9598-910fb5a106ab.jpg]

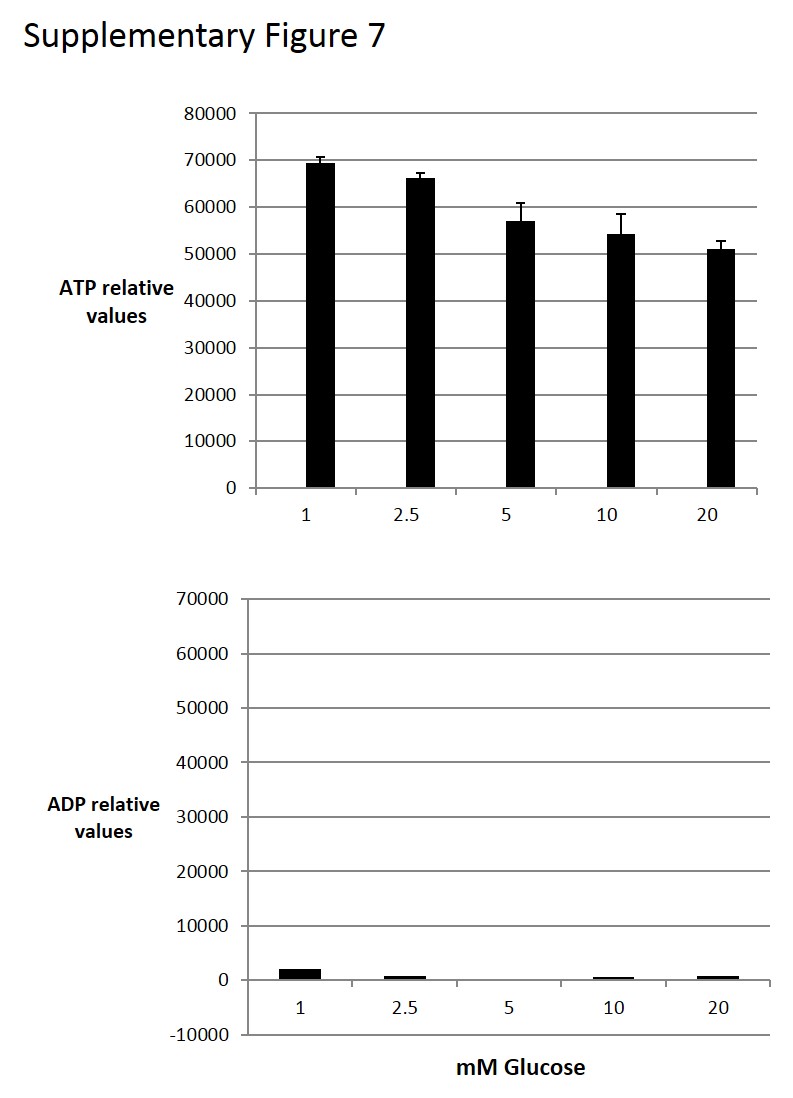

Supplement: Supplementary file 7 [file wellcomeopenres-2-16930-s0006.tgz › 0fc78e6c-a36d-4e7d-a16c-14466198d1d6.jpg]

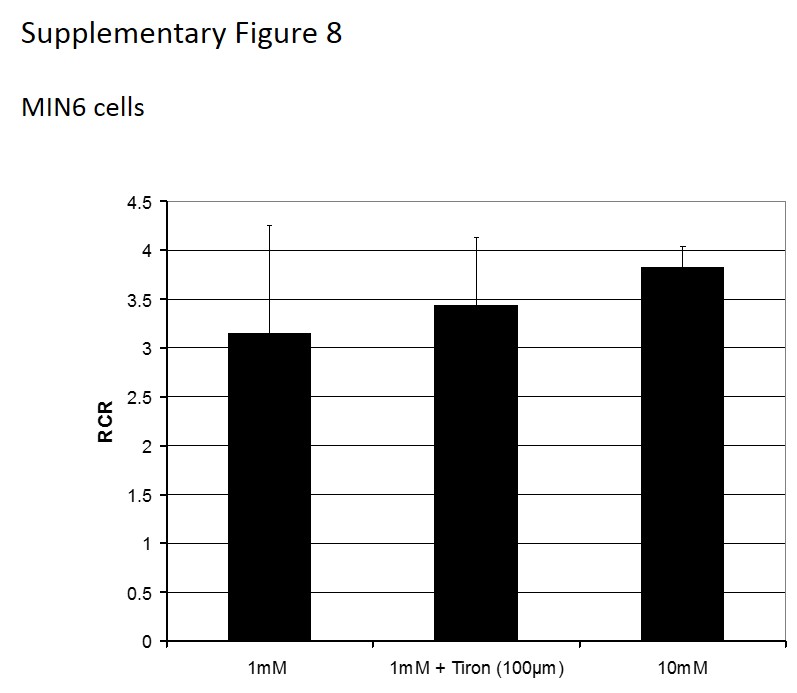

Supplement: Supplementary file 8 [file wellcomeopenres-2-16930-s0007.tgz › 1bb0860b-5a74-4cac-8892-3f267c03c25b.jpg]

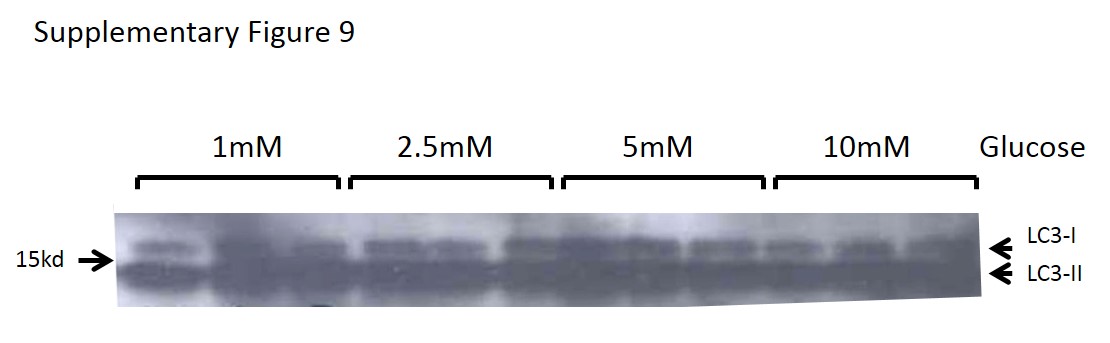

Supplement: Supplementary file 9 [file wellcomeopenres-2-16930-s0008.tgz › 0b334477-b873-4992-b11f-97cd5bcf9a31.jpg]
