# Supplementary material for: Insights into pancreatic β cell energy metabolism using rodent β cell models
Source: Wellcome Open Res. 2019 Sep 25;2:14. Originally published 2017 Feb 24. [Version 3] doi: 10.12688/wellcomeopenres.10535.3 (PMC6854877; doi:10.12688/wellcomeopenres.10535.3)
Supplement: Peer review history

# Peer review: Reviewer response for version 3

**DOI:** 10.21956/wellcomeopenres.16930.r36567

**Published:** 2019-11-13

**Authors:** Katherine E. Chapman

**Affiliations:** 1 In vitro Toxicology Group, Institute of Life Science, College of Medicine, Swansea University, Swansea, UK

I have no further comments to make.

I confirm that I have read this submission and believe that I have an appropriate level of expertise to confirm that it is of an acceptable scientific standard.

# Peer review: Reviewer response for version 2

**DOI:** 10.21956/wellcomeopenres.13719.r34379

**Published:** 2019-01-21

**Authors:** Katherine E. Chapman

**Affiliations:** 1 In vitro Toxicology Group, Institute of Life Science, College of Medicine, Swansea University, Swansea, UK

The study investigated the bioenergetic profiles of two rodent *in vitro* β-cell models in response to “high” and “low” glucose concentrations. The aim of the study was to assess the cell models’ potential for studying mitochondrial dysfunction. A thorough, multiple-endpoint analysis has been used to characterise the dose-dependent effects of glucose on the cell models used. The application of more physiologically relevant glucose concentrations than are routinely involved in *in vitro* cell culture is valuable and may be of interest to diabetes and cancer research.

 There were, however, several points identified that might improve the manuscript. These are outlined below.

1. It is appreciated that equivalent human models are currently not robust, although it would be useful to include a further justification for the particular cell lines used over other rodent insulinoma cell lines.
2. The Methods are generally sufficiently detailed. Please clarify the kits or approaches used for media glucose and lactate levels. Please also clarify the provenance of the cell lines. In the “Cell lines” section of the Methods section, it is mentioned that the cell lines were obtained from a colleague. Were these obtained directly from the other individuals mentioned who originally derived the cell lines?
3. Please could further information be included in the “Statistics” section (Methods). Were data tested for their conformation to a normal distribution to justify the use of a parametric statistical test (i.e., t-test)? If not, were data transformed? Please clarify what the error bars on graphs represent (e.g., standard deviation). Please clarify whether the experiments contributing towards “n” values (e.g., n=3) were performed as independent biological replicates on separate days.
4. Please include the sizes of SOD2 and B-actin targets for Immunoblotting (Methods) for completion. It is recommended that a labelled part of the protein standards lane would be included in the Western blot images (Figure 1).
5. In the Results section, maximum numerical value on the y-axis values should be the same within multiple graphs with the same y-axis parameters within the same figure. I would suggest setting the maximum values on these graphs to the same for both within the figure, enabling direct comparison between figures. On Figures 4 and 8, it may be helpful to indicate on the graph significant changes, as on other graphs, for consistency.
6. In Table 1, please clarify the assay used and capitalise the second M in the cell containing “1 mM” (typographical error).

I confirm that I have read this submission and believe that I have an appropriate level of expertise to confirm that it is of an acceptable scientific standard, however I have significant reservations, as outlined above.

# Peer review: Reviewer response for version 2

**DOI:** 10.21956/wellcomeopenres.13719.r26875

**Published:** 2018-04-27

**Authors:** Philip Newsholme

**Affiliations:** 1 School of Pharmacy and Biomedical Sciences, Curtin Health Innovation Research Institute (CHIRI), Curtin University, Perth, WA, Australia

The authors have adequately addressed the concerns of this referee.

I confirm that I have read this submission and believe that I have an appropriate level of expertise to confirm that it is of an acceptable scientific standard.

# Peer review: Reviewer response for version 2

**DOI:** 10.21956/wellcomeopenres.13719.r26876

**Published:** 2017-11-06

**Authors:** Charles Affourtit

**Affiliations:** 1 School of Biomedical and Healthcare Sciences, Plymouth University, Plymouth, UK

In response to a negative reception by 2 reviewers, Morten and colleagues have now made several changes to their manuscript. Its focus is still confused, however, as the authors claim different things at different places. The original version of the manuscript suggested to me quite strongly that the authors were pushing the value of insulinoma cells for studying beta cell bioenergetics. Given the unchanged title and introduction of the revised manuscript, readers could be forgiven to think that the authors are still doing so. From the rebuttals, however, it appears the authors’ true aim is to contrast insulinoma cells with non-beta-cell models. Based on comparative data, they conclude that insulinoma cells are the preferred option for studying mitochondrial activity *in vitro*, because these cells can be grown under conditions that are physiologically more relevant than the conditions needed to force other cell types to make ATP in an oxidative manner. My problem with this conclusion is that the bioenergetics of pancreatic beta cells have been characterised much less well than those of other cell types such as liver, muscle, heart, kidney and brain. From the limited data that have been reported (reviewed critically in ^1^), it is indeed becoming evident that the energy metabolism of beta cells is unusual, with regulatory and control structures that are not typically found in other cells. For *in vitro* studies on effects of drugs or genetic mutations on mitochondrial function, I would personally choose for well-established systems even though they may have to be cultured under conditions that do not fully reflect physiology. In this respect, I entirely agree with the authors that cell models are generally grown under an oxygen atmosphere and high nutritional supply that differ vastly from those prevalent *in vivo*. The attempt to mimic physiological conditions more closely is of course commendable, but the conditions applied here remain an approximation at best, since glucose and other nutrient levels faced by beta cells fluctuate continuously *in vivo*, and because physiological intracellular oxygen tensions are hard to predict. Moreover, mammalian cells are generally grown in batch cultures, which makes it impossible to precisely control the nutrient, product and gas composition of the growth medium.

 As stated above, the current manuscript could still be read as an attempt to reveal new insight in the way beta cell bioenergetics control glucose-stimulated insulin secretion (GSIS). Particularly, the authors show that the mitochondrial production of reactive oxygen species (ROS) increases after a period of glucose deprivation. The associated sensitivity of insulin release to glucose is attributed to this increased ROS. Indeed, ROS have been shown by others to induce insulin secretion, but it remains unclear to me why the elevated ROS after glucose deprivation is not enough to increase basal insulin secretion, and why glucose is still required to boost release. More generally, the presented data are correlative and, at best, offer indirect support for the authors’ claim. A different study design would be necessary to establish the relative importance of ROS and the ATP/ADP ratio as GSIS signals. As argued in my first review, the ATP levels reported here are confounded by a lack of the corresponding ADP concentrations and by compartmentalisation complications of whole-cell nucleotide measurements. In my opinion, the apparent negative effect of glucose on ATP levels should not be interpreted as violation of the classical GSIS model. Our calculation of cytosolic ATP/ADP ratios from PCr/Cr ratios yields values that are bioenergetically meaningful and indeed respond to glucose and mitochondrial uncouplers in ways that are predicted by the classical GSIS model. We will publish our data in the foreseeable future, but emphasise that the difficulty of measuring the ATP/ADP ratio that is relevant for GSIS has been discussed in detail by David Nicholls ^1^.

 My request for more detail on the applied respirometry methodology has been met, but the authors’ rebuttal is somewhat defensive. Although I indeed believe that *careful* application of Seahorse XF technology, accounting for oxygen diffusion and for medium acidification by molecules besides lactate [ref-2 ^,^^3^, offers a deeper understanding of cellular ATP fluxes than respiratory measurements alone, I wish to stress that I certainly do not suggest that this platform is a ‘one-stop shop’ for solutions to all bioenergetic problems. Indeed, complete understanding of the role of energy metabolism in GSIS cannot be obtained from metabolic fluxes alone and requires insight in relevant bioenergetic intermediates as well (see e.g. Gerencser *et al.* ^4^). Notably, our efforts to measure cytosolic ATP/ADP ratios accurately are required to quantify the relative bioenergetic control of ATP supply and ATP demand, a study that is underway. Related, I agree with the authors that measuring the intracellular oxygen tension may well offer insight in bioenergetics that obviously cannot be obtained from XF measurements. However, the data presented here do not offer such insight: a relatively low intracellular oxygen level in oxidative compared to glycolytic cells seems an inevitable consequence of differences in respiratory activity. To me, novelty cannot simply be claimed by asserting that nobody has yet published this expected consequence.

I confirm that I have read this submission and believe that I have an appropriate level of expertise to state that I do not consider it to be of an acceptable scientific standard, for reasons outlined above.

## References

[1] : The Pancreatic β-Cell: A Bioenergetic Perspective. *Physiol Rev*.2016;96(4) : 10.1152/physrev.00009.2016 1385-447 10.1152/physrev.00009.2016 27582250

[2] : Quantitative microplate-based respirometry with correction for oxygen diffusion. *Anal Chem*.2009;81(16) : 10.1021/ac900881z 6868-78 10.1021/ac900881z 19555051PMC2727168

[3] : Quantifying intracellular rates of glycolytic and oxidative ATP production and consumption using extracellular flux measurements. *J Biol Chem*.2017;292(17) : 10.1074/jbc.M116.774471 7189-7207 10.1074/jbc.M116.774471 28270511PMC5409486

[4] : Positive Feedback Amplifies the Response of Mitochondrial Membrane Potential to Glucose Concentration in Clonal Pancreatic Beta Cells. *Biochim Biophys Acta*.2017;1863(5) : 10.1016/j.bbadis.2016.10.015 1054-1065 10.1016/j.bbadis.2016.10.015 27771512PMC5398960

# Peer review: Reviewer response for version 1

**DOI:** 10.21956/wellcomeopenres.11353.r20653

**Published:** 2017-04-10

**Authors:** Philip Newsholme

**Affiliations:** 1 School of Pharmacy and Biomedical Sciences, Curtin Health Innovation Research Institute (CHIRI), Curtin University, Perth, WA, Australia

The article submitted to Wellcome Open Research by Morten KJ is a limited study investigating the ATP and ADP concentration change responses and reactive oxygen species generation, GSIS, and viability in two rodent beta cell cell lines MIN-6 and INS-1 to various glucose concentrations. The data generated and associated conclusions in the present submission are not surprising, as there is a large volume of previously published studies investigating glucose metabolism and stimulation of insulin secretion in various beta cell lines and rodent islets. The present paper does not contribute sufficient new and novel information to the to the existing literature, in the opinion of this referee.

 The various advantages and disadvantages of specific beta-cell lines have been expertly reviewed previously ^1^^,^^2^. Several lines do have high intracellular insulin content (HIT, INS-1), while others have adequate expression of GLUT2 or glucokinase (MIN6, bHC9, BRIN-BD11, CM, TRM-1). However, few are fully responsive (compared to primary islet cells) to a rise in physiological glucose with regard to insulin secretion and include INS-1, bHC, BRIN-BD11, Blox5. This is possibly because other cell lines express strong hexokinase activity, which may promote a higher sensitivity towards lower concentrations of glucose due to the low Km of the enzyme. Indeed some cell lines demonstrate either an inadequate response to glucose stimulation (MIN6, NIT-1,RINm5F, RINr, CRI-G1, In-111), or no response at all (CM, TRM-1) ^2^. Nonetheless, the most extensively studied b-cell lines are RINm5F, bTC, MIN6, HIT, INS-1 and BRIN-BD11 ^1^^,^^2^ and have aided in the understanding of pancreatic b-cell biology and functionality.

 Some of the most important advances stem from experiments investigating the impact of beta cell toxic agents including high levels of macronutrients (glucose, lipids), reactive oxygen species (ROS), and pro-inflammatory cytokines acting on b-cell viability ^3^. Equally important, are the experiments demonstrating the protective effect of specific nutrients including polyunsaturated fatty acids, amino acids and anti-oxidants ^3^. The cytoprotective action of various nutrients or other agents appear to be through modulation of the redox status of the cell toward a greater anti-oxidant potential ^4^. The lack of detail in the manuscript submitted by Morten KJ with respect to previous publications in the area is of concern and their paper is flawed until the previously published work is cited and the potential limitations of working with cell lines is fully described

 With respect to experimental design: ‘priming’ cells with a low-glucose challenge before Insulin release determination is a standard step in protocols for assaying GSIS in insulinoma cells and pancreatic islets. If cells are not deprived from glucose, they will exhibit low secretory sensitivity to glucose probably owing to an elevated basal insulin release.

I confirm that I have read this submission and believe that I have an appropriate level of expertise to state that I do not consider it to be of an acceptable scientific standard, for reasons outlined above.

## References

[1] : Nutrient regulation of beta cell function: what do islet cell/animal studies tell us?. *Eur J Clin Nutr*.2017;10.1038/ejcn.2017.4928422118

[2] : Pancreatic beta cell lines and their applications in diabetes mellitus research. *ALTEX*.2010;27(2) :105-13 2068674310.14573/altex.2010.2.105

[3] : Molecular Events Linking Oxidative Stress and Inflammation to Insulin Resistance and β-Cell Dysfunction. *Oxid Med Cell Longev*.2015;2015: 10.1155/2015/181643 181643 10.1155/2015/181643 26257839PMC4516838

[4] : Reactive oxygen and nitrogen species generation, antioxidant defenses, and β-cell function: a critical role for amino acids. *J Endocrinol*.2012;214(1) : 10.1530/JOE-12-0072 11-20 10.1530/JOE-12-0072 22547566

# Response

**Published:** 2017-09-11

**Authors:** Joanna Poulton

**Affiliations:** University of Oxford, UK

We thank the reviewer for suggesting that we discuss previous work, we look forward to citing his two reviews (his references 1 and 2) when they become available.  However these important additions do not detract from the main point of the paper, that INS-1 cells are very different from non –beta cell models that are widely used to study mitochondrial function, his comments usefully show that we should re-focus our discussion.

 We feel that he has under-estimated the novelty of our findings hence we have expanded the discussion section.  Our findings clearly show that INS1 cells could fill a unique niche for studying the toxic effects of drugs on mitochondrial function because they eliminate the need to change media glucose concentrations. To assess the effects of drugs on mitochondrial function or mitochondrial mutations canonical tumour cell lines are routinely grown in very low glucose or even galactose   For most non-beta cell lines, these conditions (“energetic stress”) force cells to use their mitochondria, being essential to demonstrate the impact mitochondrial dysfunction has on cell physiology. If experiments are carried out in high glucose media the effects of drugs reducing mitochondrial respiration can be missed  Under high glucose conditions many cells generate sufficient ATP via glycolysis to render the mitochondrial energetic impairments insignificant to cellular function.  Glucose depletion is useful in the short term but in long term studies glucose depletion imposes a significant cellular stress complicating the situation making it more difficult to determine the mode of action of a drug or mutation impacting mitochondrial function.  INS-1 cells on the other hand maintain a high rate of mitochondrial respiration on high glucose (10mM).  Hence INS1 cells are potentially an excellent model for studying the effects of mitochondrial drug toxicity because mitochondrial respiration can be constantly maintained without needing to remove glucose.

 We have added references to hexokinase as requested

# Peer review: Reviewer response for version 1

**DOI:** 10.21956/wellcomeopenres.11353.r20590

**Published:** 2017-03-10

**Authors:** Charles Affourtit

**Affiliations:** 1 School of Biomedical and Healthcare Sciences, Plymouth University, Plymouth, UK

Morten and colleagues argue that rodent insulinoma cells may provide valuable insight in the energy metabolism of pancreatic beta cells. Having studied the bioenergetics of INS-1E cells for more than a decade, I agree: mitochondrial activity in these model systems generally aligns well with the activity observed in rodent and human pancreatic islets. Indeed, cell studies from my and other labs have revealed aspects of mitochondrial ATP synthesis that appear specific (albeit not unique) to beta cells and are reproducible in islets – these aspects include a bioenergetic glucose sensitivity and relatively low coupling efficiency of oxidative phosphorylation. Notably, links between oxidative ATP synthesis defects and impaired glucose-stimulated insulin secretion (GSIS) seen under pathological conditions, are evident in both insulinoma cells and islets. In a similar spirit, INS-1 and MIN-6 cells are used here ‘(…) to determine whether they are suitable for studying the effects of mitochondrial dysfunction *in vitro*.’ This suitability is indeed becoming increasingly clear, but I do not think the data presented in this paper strengthen the case. No attempt has been made to validate any finding in primary beta cells or islets, so the physiological relevance of reported mitochondrial dysfunction remains unclear. Furthermore, the respiratory data were mostly obtained with insufficiently explained methodology and are internally inconsistent. Effects of cellular glucose deprivation on the linked respiration and insulin secretion lack novelty, whilst a glucose-induced decrease of ATP level is indeed unexpected, but most likely results from experimental limitations. On the whole, I do not feel that this paper offers the insights in beta cell energy metabolism that its title claims and, regretfully, I am unable to approve it. The following concerns are the main reason for my lack of enthusiasm.

  

1. The authors report functional effects of ‘priming’ cells with a low-glucose challenge before assays. As far as I know, a fuel deprivation of 1-2 h is a fairly standard step in protocols for assaying GSIS in insulinoma cells and pancreatic islets. As is indeed the case here, insulinoma cells are typically cultured at a glucose level (10-25 mM) that provokes insulin release. If cells are not deprived from glucose, they will exhibit low secretory sensitivity to glucose probably owing to an elevated basal insulin release. The GSIS protocol applied here detects the insulin secreted by cells incubated at various glucose levels for 30 min. However, this measurement window is preceded by a 30-min incubation at the same glucose levels – the insulin secreted during this time is discarded. Although legitimate, this unusual experimental design implies that even the cells that had not been deprived from glucose *before *the assay, were indeed fuel-limited for at least 30 min *during *the assay. From Fig. 1A it would appear that such short glucose restriction is indeed sufficient to lower the basal insulin release of INS-1 cells grown at 10 mM glucose, but fails to dampen the basal insulin secretion of MIN-6 cells that are grown at 25 mM glucose.
2. The authors report that a low-glucose priming period (a 60-min lag phase is mentioned in the legend of Fig. 3) sensitises INS-1 respiration to glucose. Again, typical protocols for insulinoma respiratory assays include incubation of cells without glucose, or at a low glucose level, before oxygen uptake measurements. It has been reported widely that mitochondrial oxidative phosphorylation of insulinoma cells as well as islets is sensitive to glucose under these conditions, and bioenergetic glucose sensitivity underpins nutrient-secretion coupling that characterises pancreatic beta cells ^1^. Although the fluorescence units make it hard to compare the INS-1 respiratory data reported in Fig. 3 with published values, and although the data have not been corrected for non-mitochondrial oxygen consumption, glucose stimulation of whole-cell INS-1 respiration is consistent with the literature and is indeed predicted by the classical GSIS model.
3. The MitoXpress assay used to measure cellular oxygen consumption is not explained very clearly and the reader is referred to previous literature for validation of the method by oxygen electrode measurements. Although cell history differs, it is concerning in this respect that the electrode data shown in Fig. 6B (no stimulation of basal respiration by 15 mM glucose) are inconsistent with the fluorescence data in Fig. 3A (large stimulation by 15 mM glucose). 
4. Similarly, the measurement of intra-cellular oxygen concentrations in cultured cells (Fig. 4) is not explained clearly, and it is hard to derive the claimed message from the way the data are presented. If I understand correctly, the authors attempt to relate intra- and extra-cellular oxygen concentrations. Such relations would be clearer from line graphs than from tables, which would facilitate comparison of different cells types. In any case, however, the author’s conclusion that intra-cellular oxygen concentration deviates more from the applied ambient oxygen tension in oxidative (e.g. INS-1) than glycolytic cells seems unsurprising.
5. An unexpected finding reported in this paper is the lowering effect of glucose on the total ATP content of ‘primed’ INS-1 cells. Although the authors rightly assert that it is the ATP/ADP ratio that is bioenergetically relevant, not the ATP level *per se*, such inverse glucose sensitivity would be at odds with the canonical GSIS model in which a glucose-induced rise in the ATP/ADP ratio triggers electrophysiological events that ultimately lead to the exocytosis of insulin-containing granules. Based on our own recent attempts, however, I suspect that this unusual finding may reflect the difficulty of measuring cytosolic ATP/ADP ratios. Indeed, the authors acknowledge explicitly they found it hard to measure accurate ADP levels, which are much lower than the ATP levels. In other words, the little ADP that is converted to ATP during the assay is to be detected against a high ATP background. We have measured ATP/ADP ratios in INS-1E cells using sulphurylase to remove ATP before ADP measurements. With this protocol we obtain total ATP/ADP ratios with values around 5-10, which increase significantly in response to glucose and collapse in the presence of a mitochondrial uncoupler (Carré J.E. and Affourtit C., unpublished). Importantly, however, interpretation of total ATP/ADP values in beta cells is confounded by multiple adenine nucleotide pools of cytosolic, mitochondrial and granular origin, respectively. Cytosolic ATP/ADP ratios can be calculated from phosphocreatine/creatine (PCr/Cr) ratios. From our recent experiments it transpired that INS-1E cells express a creatine kinase as we detected a PCr pool when cells were grown in the presence of 3 mM Cr. Assuming an equilibrium constant for the creatine kinase reaction (PCr + ADP ó Cr + ATP) of 110, we calculated a cytosolic ATP/ADP ratio of around 70, a value that increased to just below 300 in response to glucose and collapsed in the presence of uncoupler (Wynne A. and Affourtit, C., unpublished).

I confirm that I have read this submission and believe that I have an appropriate level of expertise to state that I do not consider it to be of an acceptable scientific standard, for reasons outlined above.

## References

[1] : The Pancreatic β-Cell: A Bioenergetic Perspective. *Physiol Rev*.2016;96(4) : 10.1152/physrev.00009.2016 1385-447 10.1152/physrev.00009.2016 27582250

# Response

**Published:** 2017-09-11

**Authors:** Joanna Poulton

**Affiliations:** University of Oxford, UK

We thank Dr Affourtit for supporting our conclusion that oxidative ATP synthesis defects is linked to impaired GSIS in INS-1 cells and beta cells, “…a cell type whose unique properties are important in pathogenesis” (opening sentence).   However, we did not carry out this study as a way into studying islets, interesting though this direction would be.  Instead we wish to contrast INS-1 cells with the non –beta cell models that are widely used to study mitochondrial function, hence our expanded discussion section.

 Referees comments shown in italics

*The authors report functional effects of ‘priming’ cells with a low-glucose challenge before assays. As far as I know, a fuel deprivation of 1-2 h is a fairly standard step in protocols for assaying GSIS in insulinoma cells and pancreatic islets. As is indeed the case here, insulinoma cells are typically cultured at a glucose level (10-25 mM) that provokes insulin release. If cells are not deprived from glucose, they will exhibit low secretory sensitivity to glucose probably owing to an elevated basal insulin release. The GSIS protocol applied here detects the insulin secreted by cells incubated at various glucose levels for 30 min. However, this measurement window is preceded by a 30-min incubation at the same glucose levels – the insulin secreted during this time is discarded. Although legitimate, this unusual experimental design implies that even the cells that had not been deprived from glucose before the assay, were indeed fuel-limited for at least 30 min during the assay*

 We designed our experiments to explore the impact of different times of glucose deprivation because of our initial observations that low glucose exposure increased mitochondrial ROS. We wondered if differences in levels of mitochondrial ROS linked to glucose deprivation could impact on the amount of insulin secreted in a subsequent high glucose challenge (Figure 1). The longer period of glucose deprivation increased the amount of insulin secreted at moderate glucose levels (10mM) with a decline at (15mM) compared to cells not subjected to a glucose deprivation challenge (Figure 2). As the increase in mitochondrial respiration driven by increased glucose exposure is very rapid even without a low glucose challenge (Figure 3) we suggests that the increase ROS production due to low glucose exposure could have a role in the type of GSIS response which may not be solely due to increased mitochondrial respiration and increased ATP generation. Interestingly a prolonged incubation (16hrs) in high glucose conditions 15mM eventually inhibited INS-1 oxygen consumption.

 We have now changed the order of the manuscript in order to better explain the rational of our experimental approach. Drugs that both inhibit the mitochondrial respiratory chain and generate ROS, such as rotenone can trigger insulin secretion without increasing the glucose concentration (Leloup et al (2009).  Hence the increased insulin secretion could be mediated by ROS and not just ATP. Our studies suggest that exploring the impact of mitochondrial ROS/mitochondrial signalling in the context of insulin secretion is an interesting avenue to explore and may differ from the established link to elevations of ATP driving the insulin secretion response. As the reviewer later points out the lack of a significant rise in ATP with glucose is surprising and may point to other mechanisms playing a role in the GSIS response in INS-1 cells

*From Fig. 1A it would appear that such short glucose restriction is indeed sufficient to lower the basal insulin release of INS-1 cells grown at 10 mM glucose, but fails to dampen the basal insulin secretion of MIN-6 cells that are grown at 25 mM glucose.*

 It could be speculated that the different effects observed at 10mM and 25mM glucose are due to the different effects of a short glucose restriction on the two cell lines. Min-6 cells grew less well than INS-1 cells under standard culture conditions (i.e. high glucose and 20% Oxygen). We noticed very early on that MIN-6 cell growth could be improved by culturing cells under reduced Oxygen (supplementary figure 4 B). If ROS levels are important in insulin secretion then perhaps MIN-6 cells are already primed to respond to lower concentrations of glucose. In addition to high glucose (11-25mM) used to study biology in many different types of in vitro systems Oxygen at 20% is significantly higher than that found in vivo. For example O _2_ levels around a beta cell in vivo will be likely less than 5% and could therefore have a significant impact on ROS levels and cellular metabolism. Although both referees feel this study does not add much to the current consensus as to how a beta cells function in vitro a key point to consider is how accurately the data generated from cellular in vitro β cell models and isolated pancreatic islets under non-physiological conditions accurately reflect the situation in vivo. In our opinion studies exploring the impact of substrate availability (e.g. glucose and oxygen) is of fundamental importance as we develop new models which more accurately reflect the situation in vivo. This is not only the case for the pancreatic beta cell but also neurons, stem cells and cancer cells all currently cultured in 25mM glucose with 20% oxygen.

*The authors report that a low-glucose priming period (a 60-min lag phase is mentioned in the legend of Fig. 3) sensitises INS-1 respiration to glucose. Again, typical protocols for insulinoma respiratory assays include incubation of cells without glucose, or at a low glucose level, before oxygen uptake measurements. It has been reported widely that mitochondrial oxidative phosphorylation of insulinoma cells as well as islets is sensitive to glucose under these conditions, and bioenergetic glucose sensitivity underpins nutrient-secretion coupling that characterises pancreatic beta cells ^1^. Although the fluorescence units make it hard to compare the INS-1 respiratory data reported in Fig. 3 with published values, and although the data have not been corrected for non-mitochondrial oxygen consumption, glucose stimulation of whole-cell INS-1 respiration is consistent with the literature and is indeed predicted by the classical GSIS model.*

 It is true that other systems such as the Sea Horse bioanalyser convert the values from a fluorescence detection system into nmoles of O _2_ allowing rates of oxygen consumption to be given units with rates calculated over time. A similar detection system is used with the Luxcel MitoXpress probes with the signal quenched by the presence of Oxygen. In Figure 3 we use fluorescence values as an indicator of Oxygen levels in the oil sealed well and form this data calculate a rate of change of fluorescence which reflects the rate of oxygen consumption. In figure 3 we are primarily comparing the difference in oxygen consumption of the two lines rather than making a comparison with other publications on INS-1 cells. Therefore in the context of this paper we feel our conclusions are valid. The reason rates of oxygen consumption in nmoles of O _2_ are not calculated with mitoxpress xtra is that we are concerned about differences in back diffusion as oxygen is removed from the sealed well. Oxygen can diffuse through the plastic plate and through the oil. If this occurs at different rates when O _2 _levels are at 15% and 5% respectively this will affect the respiration rate which should be adjusted to account for the rate of back diffusion. As we do not know the levels of back diffusion then we cannot accurately give a rate of O _2 _consumption per unit time. A similar issue is observed with the Sea Horse system with back diffusion around the sealing probe as the well is sealed with a plunger to make the O _2 _measurements. As the seal may vary within wells in a plate and between plates the oxygen concentration values are unlikely to 100% accurate. The fast rate of oxygen consumption of the INS-1 cell line in high glucose media is confirmed in figure 4 using the intracellular O _2_ probe. Here fluorescence life time rather than fluorescence values are used with an O _2 _calibration curve to accurately measure intracellular O _2_ levels. Intracellular O _2_ levels are much lower than the ambient O _2_ levels even when ambient O _2_ levels are low further suggesting a high rate of mitochondrial respiration. As shown in figure 4 Antimycin A intracellular O _2_ levels for INS-1 closely reflect the ambient oxygen levels of the plate reader supporting the idea that mitochondrial respiration in thus line is the primary source of cellular oxygen consumption.

*The MitoXpress assay used to measure cellular oxygen consumption is not explained very clearly and the reader is referred to previous literature for validation of the method by oxygen electrode measurements.*

 While less widely used and marketed than Seahorse, we assert that MitoXpress is well validated.  We concede that it is less well known and have therefore added the explanation requested as an extra supplementary figure (see supplementary figure 3).

*Although cell history differs, it is concerning in this respect that the electrode data shown in Fig. 6B (no stimulation of basal respiration by 15 mM glucose) are inconsistent with the fluorescence data in Fig. 3A (large stimulation by 15 mM glucose).* 

 The cells used in Fig 6 would have been grown for a prolonged period > 16hrs in 15mM glucose before O _2_ rates were determined. Those in Fig 3 A were measured straight away with the O _2_ rate measured within 60 minutes of glucose addition. We have now made this clear in the text. A discussion point has also been added which comments on the potential differences of short and long term 15mM glucose exposure on INS-1 cells in the context of other cancer cell lines. Not only does high glucose exposure reduce mitochondrial respiration in other cancer cell lines following long term exposure it is a concentration 5mM higher than the RPMI media used to grow INS-1 cells. There is the potential for high glucose exposure to impact on INS-1 function which could reduce levels of mitochondrial respiration

*Similarly, the measurement of intra-cellular oxygen concentrations in cultured cells (Fig. 4) is not explained clearly, and it is hard to derive the claimed message from the way the data are presented.*

 The calibration curve and subsequent equation are shown in Supplementary figure 2 and this is referred to more clearly in the text with further explanation of how the probe works in a new supplementary figure.  Supplementary figure 3 shows the type of intracellular oxygen data generated at a single O _2_ concentration.

*If I understand correctly, the authors attempt to relate intra- and extra-cellular oxygen concentrations. Such relations would be clearer from line graphs than from tables, which would facilitate comparison of different cells types. In any case, however, the author’s conclusion that intra-cellular oxygen concentration deviates more from the applied ambient oxygen tension in oxidative (e.g. INS-1) than glycolytic cells seems unsurprising.*

 Although we have taken key O _2_ values at specific oxygen concentrations from the curves to generate the tables. The curves shown giving intracellular oxygen levels in the presence of antimycin A show the actual data and are indicated on the graphs on Figure 4.Maybe unsurprising to this reviewer but novel given that no one has previously published on this. Furthermore the switching between oxidative and glycolytic metabolism might affect intracellular O _2_ conc and hence signals such as HIF activation is widely overlooked.  We have therefore expanded this in the discussion section. We assert that the mitoxpress intra probe will be an important tool in future research into cell metabolism and bioenergetics. The probe can be used in real time assays over hrs or days and be multiplexed with other assays include JC1 (Caymen) and cell tox green (Promega) in plate based assays.  In this it offers significant advances over the Sea Horse system. Figure 4 in the manuscript was obtained by loading cells with Mitoxpress intra and then measuring intracellular oxygen levels with cells grown in high glucose media at different O _2_ levels. The glycolytic line U87MG behaves very differently in low glucose media where cells switch to mitochondrial respiration Potter et al (2016).

*An unexpected finding reported in this paper is the lowering effect of glucose on the total ATP content of ‘primed’ INS-1 cells. Although the authors rightly assert that it is the ATP/ADP ratio that is bioenergetically relevant, not the ATP level per se, such inverse glucose sensitivity would be at odds with the canonical GSIS model in which a glucose-induced rise in the ATP/ADP ratio triggers electrophysiological events that ultimately lead to the exocytosis of insulin-containing granules.*

 We are pleased that the reviewer finds this key finding intriguing. The cells in the reduced glucose concentration also show reduced lactate production which could be indicative of a reduce glycolysis with the cells moving into a quiescent state. Under the low glucose conditions where cells appear to be carrying out less glycolysis (i.e. reduced lactate) other substrates could be utilised by mitochondria as a fuel. As growth rates drop markedly in INS-1 cells when cultured in reduced glucose media see supplemental figure 3 the reduced growth and ATP generated from non-glucose substrates could result in a net increase in total ATP. The net increase in ATP observed with short low glucose exposure compared to high (20mM) (1hr) shown in figure 5 is also observed after 24hrs (data not shown).

*Based on our own recent attempts, however, I suspect that this unusual finding may reflect the difficulty of measuring cytosolic ATP/ADP ratios.*

*Indeed, the authors acknowledge explicitly they found it hard to measure accurate ADP levels, which are much lower than the ATP levels. In other words, the little ADP that is converted to ATP during the assay is to be detected against a high ATP background. We have measured ATP/ADP ratios in INS-1E cells using sulphurylase to remove ATP before ADP measurements. With this protocol we obtain total ATP/ADP ratios with values around 5-10, which increase significantly in response to glucose and collapse in the presence of a mitochondrial uncoupler (Carré J.E. and Affourtit C., unpublished). Importantly, however, interpretation of total ATP/ADP values in beta cells is confounded by multiple adenine nucleotide pools of cytosolic, mitochondrial and granular origin, respectively. Cytosolic ATP/ADP ratios can be calculated from phosphocreatine/creatine (PCr/Cr) ratios. From our recent experiments it transpired that INS-1E cells express a creatine kinase as we detected a PCr pool when cells were grown in the presence of 3 mM Cr. Assuming an equilibrium constant for the creatine kinase reaction (PCr + ADP ó Cr + ATP) of 110, we calculated a cytosolic ATP/ADP ratio of around 70, a value that increased to just below 300 in response to glucose and collapsed in the presence of uncoupler (Wynne A. and Affourtit, C., unpublished).*

 This is an important point as we have had problems using ATP/ADP kits using other models (i.e. primary rat foetal neurons). If the Affouritt data is published we would be glad to reference it in the paper as the problems using these ATP/ADP ratio assays needs to be pointed out and alternative approaches highlighted. Changes in AMP which are larger than those for ADP and ATP maybe a better indicate of metabolic switching as cells transition from one carbon source to another.

**References**

 Potter M (2016) The Warburg effect: 80 years on Biochem Soc Trans. 2016 Oct 15; 44(5): 1499–1505.

Zhdanov AV (2015) Differential contribution of key metabolic substrates and cellular oxygen in HIF signalling. Exp Cell Res. 2015 330(1):13-28. doi: 10.1016/j.yexcr.2014.10.005.

 Leloup C (2009­) Mitochondrial reactive oxygen species are obligatory signals for glucose-induced insulin secretion. *Diabetes.* 2009; **58**(3): 673–81
